# Nonlinear Theta-Gamma Coupling between the Anterior Thalamus and Hippocampus Increases as a Function of Running Speed

**DOI:** 10.1523/ENEURO.0470-21.2023

**Published:** 2023-03-15

**Authors:** Yu Qin, Alex Sheremet, Tara L. Cooper, Sara N. Burke, Andrew P. Maurer

**Affiliations:** 1 Engineering School of Sustainable Infrastructure and Environment, University of Florida, Gainesville, FL 32611; 2 McKnight Brain Institute, Department of Neuroscience, University of Florida, Gainesville, FL 32610; 3 Department of Biomedical Engineering, University of Florida, Gainesville, FL 32611

**Keywords:** cross-frequency coupling, limbic, mouse, Papez

## Abstract

The hippocampal theta rhythm strongly correlates to awake behavior leading to theories that it represents a cognitive state of the brain. As theta has been observed in other regions of the Papez circuit, it has been theorized that activity propagates in a reentrant manner. These observations complement the energy cascade hypothesis in which large-amplitude, slow-frequency oscillations reflect activity propagating across a large population of neurons. Higher frequency oscillations, such as gamma, are related to the speed with which inhibitory and excitatory neurons interact and distribute activity on the local level. The energy cascade hypothesis suggests that the larger anatomic loops, maintaining theta, drive the smaller loops. As hippocampal theta increases in power with running speed, so does the power and frequency of the gamma rhythm. If theta is propagated through the circuit, it stands to reason that the local field potential (LFP) recorded in other regions would be coupled to the hippocampal theta, with the coupling increasing with running speed. We explored this hypothesis using open-source simultaneous recorded data from the CA1 region of the hippocampus and the anterior dorsal and anterior ventral thalamus. Cross-regional theta coupling increased with running speed. Although the power of the gamma rhythm was lower in the anterior thalamus, there was an increase in the coupling of hippocampal theta to anterior thalamic gamma. Broadly, the data support models of how activity moves across the nervous system, suggesting that the brain uses large-scale volleys of activity to support higher cognitive processes.

## Significance Statement

Theta and gamma are the local-field potential (LFP) rhythms often studied in the hippocampal and entorhinal areas. However, these brain regions are only a component of the reentrant anatomy of the limbic system, suggesting that oscillatory interactions may reflect a more global process of neural organization. Here, we report theta and gamma interactions between hippocampus and the anterior dorsal and ventral nuclei of the thalamus that increase in strength as a function of running speed. These data reinforce the theory that larger rhythms are the physiological consequence of large-scale synaptic events across brain regions, where smaller oscillations represent the activity of a smaller synaptic pool.

## Introduction

Interactions between the thalamus and the hippocampus are thought to support emotional expression (Papez, 1995), learning, memory, and spatial navigation (O’Keefe and Nadel, 1978; Aggleton and Sahgal, 1993; Aggleton et al., 1996; Byatt and Dalrymple-Alford, 1996; Vann et al., 2000; Fortin et al., 2002; Vann and Aggleton, 2004; Clark and Taube, 2012). Within the Papez circuit, the subiculum strongly projects to the anterior thalamus through the postcommissural fornix (Ishizuka, 2001; Wright et al., 2010), potentially responsible for the entrainment of anterior thalamic neurons to theta (∼8 Hz; Albo et al., 2003; Tsanov et al., 2011a, b; Pastor et al., 2020). This anatomic projection supports the hypothesis that theta “courses” through the entire Papez circuit (Vertes et al., 2001; McNaughton and Vann, 2022), supporting the phasic reentry hypothesis in which neural activity is coordinated and reinforced via the return of activity through recurrent projections (Hebb, 1958; Edelman and Mountcastle, 1978; Edelman, 1987; Skaggs, 1995; von Stein and Sarnthein, 2000).

Buzsáki and Draguhn proposed the energy cascade (Buzsáki and Draguhn, 2004; Buzsáki, 2006). Larger loops support low frequency-high amplitude rhythms, while activity distributed into smaller, nested local loops gives rise to lower amplitude, faster rhythms. The energy cascade hypothesis shares similarities with the classical physics description of turbulence (Sheremet et al., 2018a; Deco and Kringelbach, 2020). The most straightforward metaphor to convey the idea that nested loops can be related to interdependent oscillations is perhaps the phrase:

*Big whorls have little whorls*

*Which feed on their velocity*

*And little whorls have lesser whorls*

*And so on to viscosity* (Richardson, 2007)

The large reentrant loops (“big whorls”) that support theta also provide the excitatory input that drives the nested, smaller population of excitatory and inhibitory neurons (“little whorls”), giving way to short bursts of high-frequency activity such as the gamma rhythm (Buzsáki and Wang, 2012). As hippocampal theta power increases with running speed (Whishaw and Vanderwolf, 1973; Morris and Hagan, 1983), it can be anticipated that more activity is pushed into smaller loops, where faster frequency rhythms (e.g., gamma) increase in amplitude with higher running speeds (Chen et al., 2011; Ahmed and Mehta, 2012; Kemere et al., 2013; Zheng et al., 2015). The gamma rhythm has been hypothesized to arise from perisomatic inhibition (Mann et al., 2005; Hájos and Paulsen, 2009), with hippocampal basket cells simultaneously bursting with theta and gamma rhythmicity (Buzsáki et al., 1983; Bragin et al., 1995). Given the nested nature of anatomic loops and that cells are modulated by both theta and gamma, it can be expected that hippocampal theta-gamma cross-frequency coupling increases as a function of running speed (Sheremet et al., 2019).

However, the hippocampus is only one region in a larger circuit. As the hippocampus has significant projections to the anterior thalamus, the two areas should have strong theta-theta coupling. Moreover, gamma-band activity in the anterior thalamus should be coupled to activity in the hippocampus. One potential source of fast local rhythmicity may be a voltage dependent oscillation as seen in the reticular thalamus (Pinault and Deschênes, 1992). However, as inhibitory interneurons and the GABA_A_ receptors are often related to gamma coordination (for extensive review, see Buzsáki, 2006), it is plausible that inhibitory interneurons may also play a role. To our knowledge, the anterior thalamus does not have local interneurons (Wang et al., 1999). Rather, the prominent source of inhibition into the anterior thalamus theoretically arises from the reticular thalamus (Halassa and Acsády, 2016). Specifically, there is evidence that the presubiculum provides a form of feed-forward inhibition to the anterior thalamus via the reticular thalamus (Vantomme et al., 2020), facilitating the timing of activity flow through the Papez circuit and sharpening the timing of head-direction neurons in the ADN (Vantomme, 2020). Another potential source of inhibitory input may arise from long-range CA3 interneuron projections (Vetere et al., 2021). This begs the question if inhibition is responsible for gamma and yet the interneurons coordinating the dynamic are outside of the population, is there significant gamma in the anterior thalamus, and is there cross-frequency coupling? To address this, we analyzed data from prior publications that simultaneously recorded from the hippocampus and anterior dorsal and anterior ventral thalamus in freely-behaving mice (Peyrache et al., 2015b, 2017; Viejo and Peyrache, 2020). We found that, although gamma was an order of magnitude lower in the anterior thalamus relative to the hippocampus, as running speed and theta power increased, there was greater cross-regional theta and gamma coupling between the hippocampus and anterior thalamus.

## Materials and Methods

### Analytical database

For the present study, we analyzed data from four mice downloaded from the Collaborative Research in Computational Neuroscience data sharing website (https://portal.nersc.gov/project/crcns/download/th-1/data). These data were generously provided by the Peyrache and Buzsáki laboratories (Peyrache et al., 2015a). The mice used in this manuscript have been analyzed with different approaches and analytical questions in mind (Peyrache et al., 2015a,b, 2017; Viejo and Peyrache, 2020). As we were interested in hippocampal-thalamus interactions, we selected the following datasets: Mouse12-120809, Mouse17-130129, Mouse32-140822, and Mouse20-130515. For M17, M20, and M32, the behavior was foraging in an open environment, while M12 behavior was on a radial arm maze.

### Time-series analysis

#### Stochastic Fourier analysis, spectrum and (cross-)bispectrum

We decomposed LFP time series using the discrete Fourier transform (DFT), under the assumption that they represent realizations of a stochastic process, stationary in the relevant statistics. Let p(t) be a stochastic process and $p_{j}=p\left(t_{j}\right)$ one realization of the process sampled at Δt time increments, with $P_{n}$ its DFT pair (Weaver, 1989), defined by the equation

Pn=∑j=0N−1pj exp(−2πfntj);pj=1N∑n=0N−1Pn exp(2πfntj);tj=jΔt;Δfn=nΔf

$$with\text{ Δ}f=\frac{1}{N\text{Δ}t},and\;j,n=0,2,...,N-1,$$

where N is the number of points of the discretization, and sequences ${\left\{t_{j}\right\}}_{1,N}$ and ${\left\{f_{n}\right\}}_{1,N}$ are sometimes called the time and frequency grids. If $P_{n}$, $Q_{n}$, and $R_{n}$ are the DFT of time sequences $p_{j}$, $q_{j}$, and $r_{j}$, regarded as realizations of distinct stochastic processes, the cross-spectrum and cross-bispectrum estimators are defined as

(1) $$S_{n}^{pq}=S^{pq}\left(f_{n}\right)=\langle P_{n}Q_{n}^{*}\rangle$$

(2) $$B_{mn}^{pqr}=B^{pqr}\left(f_{m},f_{n}\right)=\langle P_{m}Q_{n}R_{m+n}^{*}\rangle ,$$

where the angular brackets denote the ensemble average, and the asterisk denotes complex conjugation. If a single process is involved, S and B are usually called “spectrum” and “bispectrum,” respectively. The “auto”-spectrum, commonly referred to simply as the “spectrum,” is the degenerate form of the cross-spectrum, $S_{n}^{pp}$ with p≡q.

The cross-bispectrum estimate is typically used in the “auto” form (also called “bispectrum”), i.e., for a single stochastic process $B_{mn}^{ppq}$ (or simply as $B_{mn}^{p}$), and normalized as

(3) $$b_{mn}^{p}=\frac{B_{n,m}^{p}}{{\left(\left\langle {\left|P_{n}P_{m}\right|}^{2}\rangle \langle {\left|P_{n+m}\right|}^{2}\right\rangle \right)}^{1/2}}.$$

The normalization used in Equation 3 ensures that $\left|b_{mn}^{p}\right|\leq 1$ (Haubrich and MacKenzie, 1965; Elgar and Guza, 1985). With a slight abuse of terminology, will refer to the modulus $\left|b_{mn}^{p}\right|$ and the phase $\arg\left(b_{mn}^{p}\right)$ are as bicoherence and biphase, respectively (the bicoherence is commonly defined as ${\left|b_{mn}^{p}\right|}^{2}$).

Using the Hermitian property of the DFT of real sequences $P_{-n}=P_{N-n}=P_{n}^{*}$, it easy to show that the cross-bispectrum of two stochastic processes p and q, that the cross-bispectrum has the following symmetries: (1) $B_{-m,-n}^{ppq}=B_{mn}^{ppq}$; (2) $B_{mn}^{ppq}=B_{nm}^{ppq}$; and (3) $B_{s_{1}m,s_{2}n}^{ppq}=B_{-s_{1}m,-s_{2}n}^{ppq}$, where $s_{1,2}=\pm$ . If the cross-bispectrum is represented in a plane with the axes defined by $f_{m}$ and $f_{n}$, these symmetries imply that (1) quadrants 1–3 and 2–4 are equivalent, (2) semiplanes separated by the first diagonal are equivalent, and (3) semiplanes separated by the second diagonal are equivalent, and, consequently, the principal (nonredundant) domain of cross-bispectra of the $B_{mn}^{ppq}$ type is given by quadrants 1 and 8 in the plane, bounded by the maximum frequency $f_{N}$ . For further information on how to interpret these figures, please see Sheremet et al. (2020). The bispectrum $B_{mn}^{ppp}$ has the additional symmetry $B_{m,-n}^{ppp}=B_{m-n,n}^{ppp}$, which implies that octants 1 and 8 are also equivalent, and therefore the principal domain reduces in this case to octant 1, bounded by $f_{N}$.

The bispectrum arises naturally in relation to the non-Gaussian character of the process p(t): the real part of normalized bispectrum $ℜ\left\{b_{mn}^{p}\right\}$is related to the skewness of the time sequence $p_{j}$, while its imaginary part, $I\left\{b_{mn}^{p}\right\}$, is related to the asymmetry of $p_{j}$ (Haubrich and MacKenzie, 1965; Masuda and Kuo, 1981). Cross-bispectra may be used to quantify the phase coupling across distinct time series (for example, LFP traces recorded in different parts of the brain). However, a direct interpretation in terms of skewness and asymmetry is not available in this case. The use of cross-bispectra was first proposed to describe the third-order statistics of ocean waves (Hasselmann et al., 1963). Bispectral analysis has a wide application in nonlinear systems, ranging from water waves, large scale interplanetary scintillation, plasma turbulence, to small-scale pitch detection, image reconstruction, and machine fault diagnosis (see, Elgar and Guza, 1985; Chiang and Nikias, 1990; Spicher et al., 2015; Itoh et al., 2017). Cross-bispectrum analysis has been applied to study nephrons of the kidney (Siu and Chon, 2009) and electroencephalography (EEG) (Isler et al., 2008).

The use of bispectrum in analyzing electroencephalography (EEG) can be traced back to Kleiner et al. (1969). In fact, to account for the nonsinusoidal nature of the hippocampal theta rhythm, Walter and Adey (1963) suggested bispectral estimation over five decades ago. Because of the inherent nonlinearity of EEG signal, bispectral analysis has been applied to qualify the nonlinear coupling in different brain regions and under different physiological states (Bullock et al., 1997; Andrzejak et al., 2001). Bispectral analysis was used by Sheremet et al. (2016) to investigate the evolution of the nonlinear character of the hippocampal LFP as a function of rat running speed. An in-depth discussion of the bispectral estimator in relation to the nonlinear coupling estimators popular in neuroscience can be found in previously published work (Kovach et al., 2018).

Following Sheremet et al. (2019), the spectral and bispectral analysis performed here focuses on LFP epochs in which the dominant order parameter is running speed. Cross-spectra were estimated using the Welch method (Welch, 1967; Watts and Jenkins, 1968). Cross-bispectra were computed using codes based on modified functions of the HOSA toolbox (Swami et al., 2003). LFP time series were de-meaned, and divided into 50% overlapping segments of 1250-point windows for the sampling rate of 1250 Hz, with a frequency resolution of ∼1 Hz. The number of degrees of freedom of the spectral estimators across varies across rats and as a function of speed. A DOF of the order of 100 is typical for most speed levels with an overall significance level |b|≳0.1. All calculations were coded in MATLAB, using its implementation of the DFT.

#### Detection of power correlations

We implemented the method of calculating the correlation coefficients of the spectrogram as outlined by Masimore et al. (2004, 2005), which allows the fundamental frequencies of the LFP to be identified without filtering as well as determine any potential interactions across different oscillatory bands. The power cross-correlograms were obtained by estimating the correlation coefficients between all the frequency pairs in the output of the DFT. It should be noted here that, as the variance of both the theta and gamma rhythm (as well as their interaction) depends on velocity, all power spectra across all running speeds were included in this analysis (Sheremet et al., 2018a, 2019).

#### Investigation of Head direction and theta phase selectivity

To determine head direction or theta phase selectivity for single neurons, spike timings were interpolated with mouse head direction or theta phase in a temporal resolution of 1250 Hz. Directions or phases (0°–360°) were evenly divided into 12-degree bins (i.e., 0°−30°; 30°−60°;…330°−360°). Values were occupancy normalized by the amount of time spent in each bin. Head direction modulation depth or theta modulation depth was derived by calculating the normalized variation of firing rate across degree bins, i.e., (max frequency − min frequency)/(max frequency) of the bin-wise firing rates. For each cell, we also performed a Rayleigh z-test between the bin-wise firing rates and bins. We regarded the cells that (1) have a nonuniform distribution of “phase-wise” firing rates with a probability >0.995 and (2) have at least 0.1 modulation depth as theta modulated neurons; In order to determine head direction selectivity, we used the method as reported previously (Peyrache et al., 2015b). Cells were classified as head-direction modulated if they were found to (1) have a nonuniform distribution of “directional” firing rates with a probability >0.995 and (2) have at least 1.0 concentration of distribution of “directional” firing rates as HD cells. Then we plotted theta phase modulation depth and HD modulation depth versus averaged firing rate for each cell (see Results).

#### Calculation of power spectral densities on spike trains

To calculate the power spectral density of spike trains of individual cells sorted by running speed, spike timing records were first interpolated with mice position records in a temporal resolution of 1250 Hz. Then the time series were separated into segments of 1 s. Speed records were obtained for each segment by calculating the time derivative of position data, and then the segments were sorted into velocity bins by the averaged speed of each segment.

For each velocity bin, time-series segments of spikes were converted into binary data (1250 Hz) and then spectrally decomposed for individual cells to examine the burst frequency modulation (Leung and Buzsáki, 1983; Sheremet et al., 2016; Zhou et al., 2019). To ensure that a high firing rate neuron does not skew the overall results, each cell was normalized by mean power, resulting in power being presented in arbitrary units. The cells were sorted into three categories: HD cells (passed Rayleigh z-test and concentration of directional distribution > 1), theta phase cells (passed Rayleigh z-test with a phase modulation depth >0.1), and other cells (neither HD cells nor theta phase cells). The results are shown in the Results. Note that the vertical axis is organized by the mean firing rate of individual cells, and the averaged power spectra of the cell population are presented to the right.

## Results

**Figure 1.**
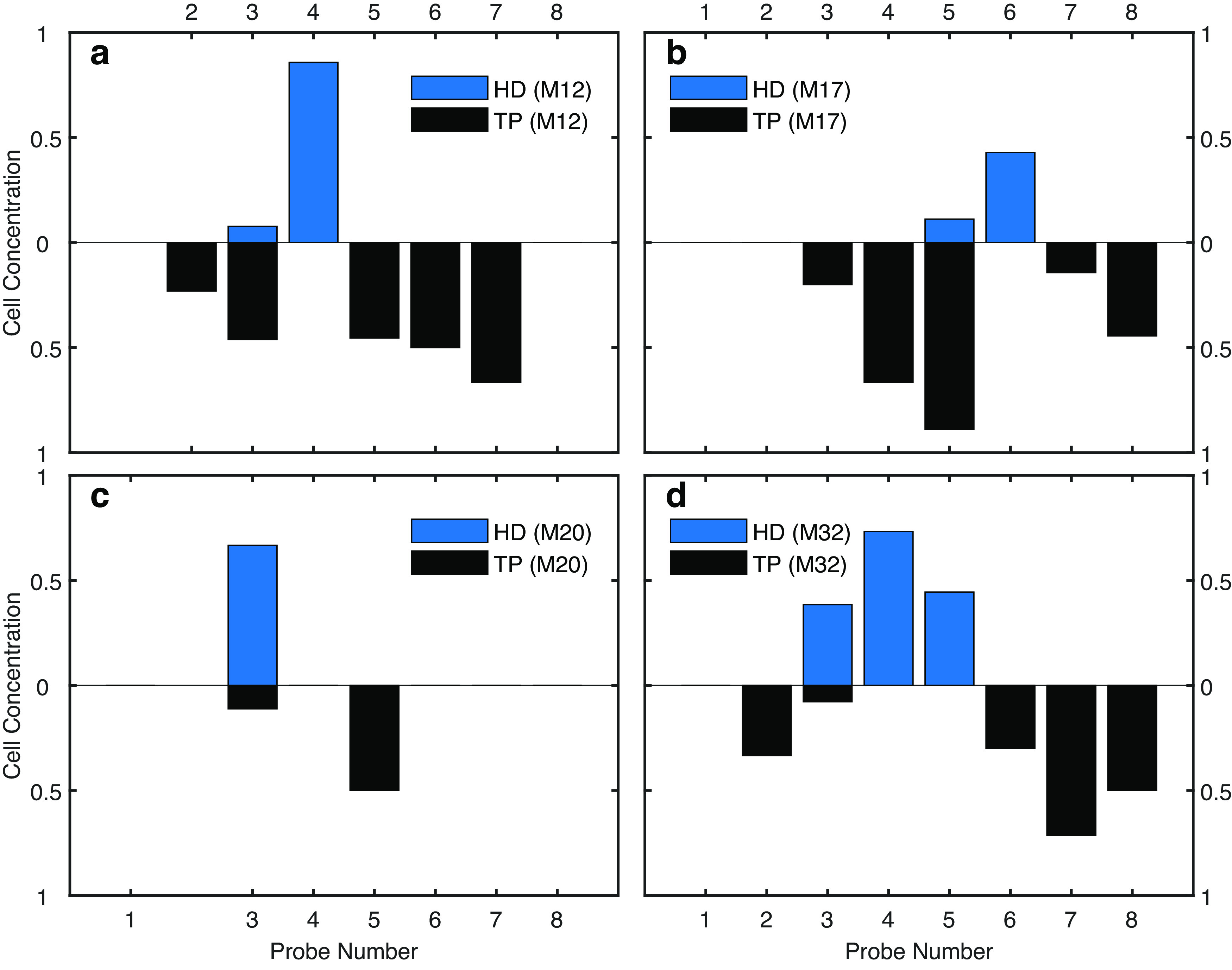
The concentration of head direction cells and theta phase cells as a function of electrodes in anterior thalamus for all the mice. Note that the missing horizontal ticks are broken probes. The Total number of cells is *N* = 184. We use the following probe numbers to estimate the anterior thalamus subregions: (***a***) M12: ADT-Probe 4, AVT-Probe 7; (***b***) M17: ADT-Probe 6, AVT-Probe 8; (***c***) M20: ADT-Probe 3, AVT-Probe 5; (***d***) M32: ADT-Probe 4, AVT-Probe 7.

Localization of electrodes in the thalamic regions is built on prior work (Peyrache et al., 2015b), where thalamic probes were implanted in the left hemisphere, perpendicularly to the midline (AP: –0.6 mm; ML: –0.5 to –1.9 mm; DV: 2.2 mm), with a 10°–15° angle, the shanks pointing toward midline (Peyrache et al., 2015b; see their Supplementary Fig. 1a-f). The 8-shank probe, Neuronexus Buz64 design, was a linear array implanted medio-laterally, and that AV lies latero-ventrally to AD (Peyrache et al., 2015b; see their Supplementary Fig. 1c). To reinforce the localization of electrodes within the anterior thalamus, The proportion of head direction and theta modulated neurons was plotted across the 8-shank silicon probe (Fig. 1). The most medial shank with the highest concentration of head direction modulated cells was selected as the ADT, while the most lateral shank with the highest proportion of theta modulated neurons was selected as the AVT representative.

**Figure 2.**
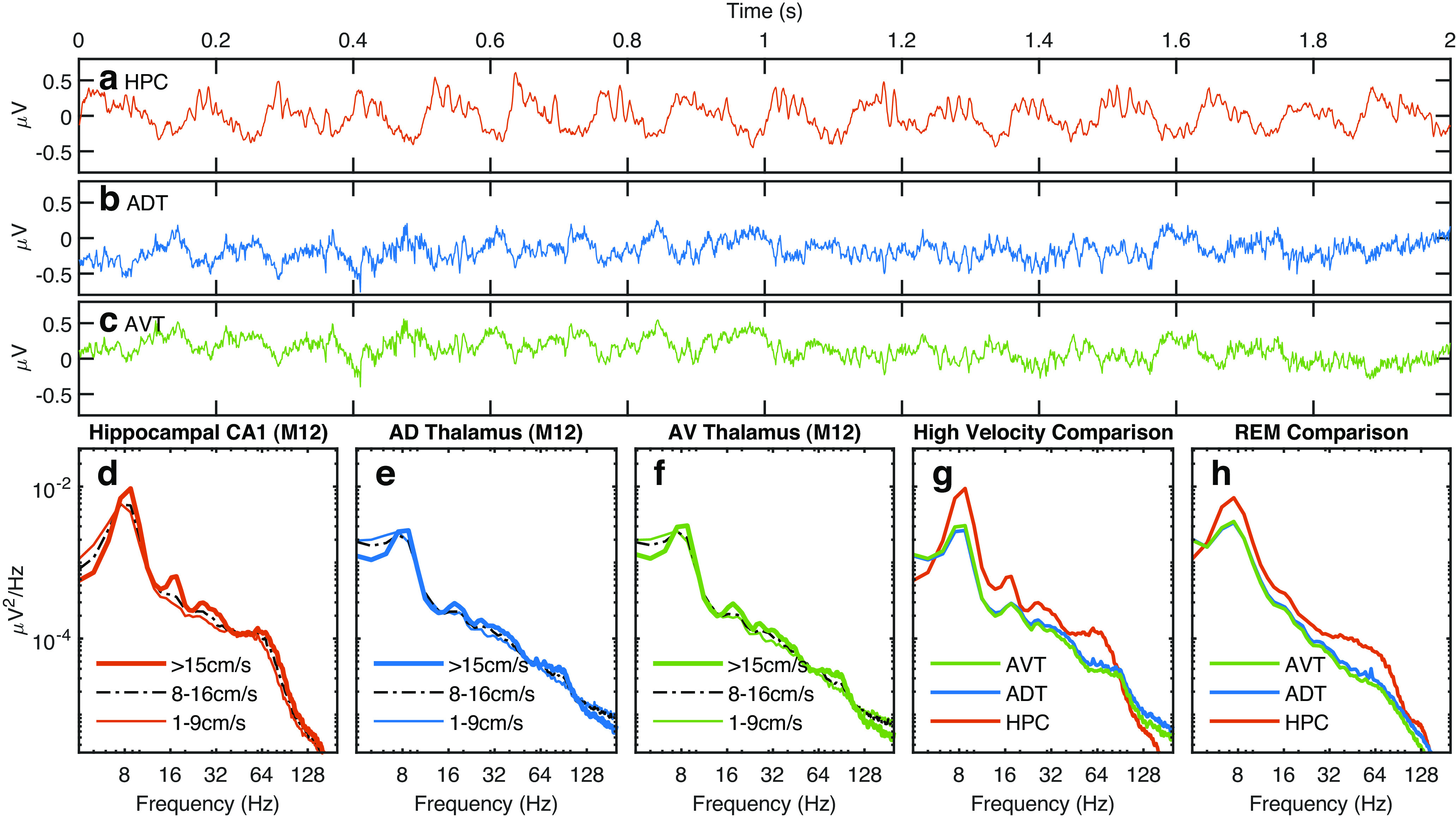
Example LFP traces and power spectral density of LFP in hippocampal CA1 region and anterior thalamus subregions as a function of velocity. ***a***, Two-second trace of the local-field potential from the mouse CA1 layer, (***b***) anterior dorsal thalamus, and (***c***) anterior ventral thalamus. ***d***, The hippocampal CA1 region exhibits a prominent gamma deviation above the slope. ***e***, ***f***, The anterior thalamus appears to show a more linear power law of the type where the amplitude is equal to the reciprocal of frequency, while the multislope slope nature of the power spectra (Sheremet et al., 2018a) becomes evident in hippocampal CA1 with a steeper slope at ∼80 Hz and more overall power. At running speeds of >15 cm/s or, there are multiple prominent harmonics of theta which approach 32 Hz in both CA1 and anterior thalamus. ***g***, ***h***, While there is comparable power in the theta range between the CA1 region and the anterior thalamus, there is less gamma in the anterior thalamus, in addition, REM features less theta and theta harmonics but a wider bandwidth of the gamma bump in CA1. (data from M12).

We then examined the power spectral density of the local field potential (LFP) in CA1 area of the hippocampus and anterior thalamus subregions of mice as a function of running speed (Fig. 2). From the power spectral density, the hippocampus and both anterior thalamus subregions exhibited a strong theta rhythm during running (Fig. 2*a–c*). At either the low or high running speeds, the anterior thalamus expressed a typical amplitude = 1/frequency linear relationship when plotted on a log-log scale, whereas the hippocampal power spectra was multisloped (Sheremet et al., 2018a), with the primary difference being that the hippocampus had higher power in the 6–110 Hz frequency range. Previously, we have described this frequency range in the rat hippocampus as being occupied by theta, the harmonics of theta, and a unitary gamma band (60–120 Hz; Sheremet et al., 2016, 2019). In the hippocampus and anterior thalamus, there was an increase in prominence in the 8–10 Hz band at running speed >15 cm/s relative to 1–9 cm/s. In accord with the idea that there is also an increase in theta harmonics with running speed, power also increased in adjacent frequencies, which could potentially correspond to the 16- to 20-, 24- to 30-, and 32- to 40-Hz bands with integer relationships to the fundamental 8- to 10-Hz frequency band.

**Figure 3.**
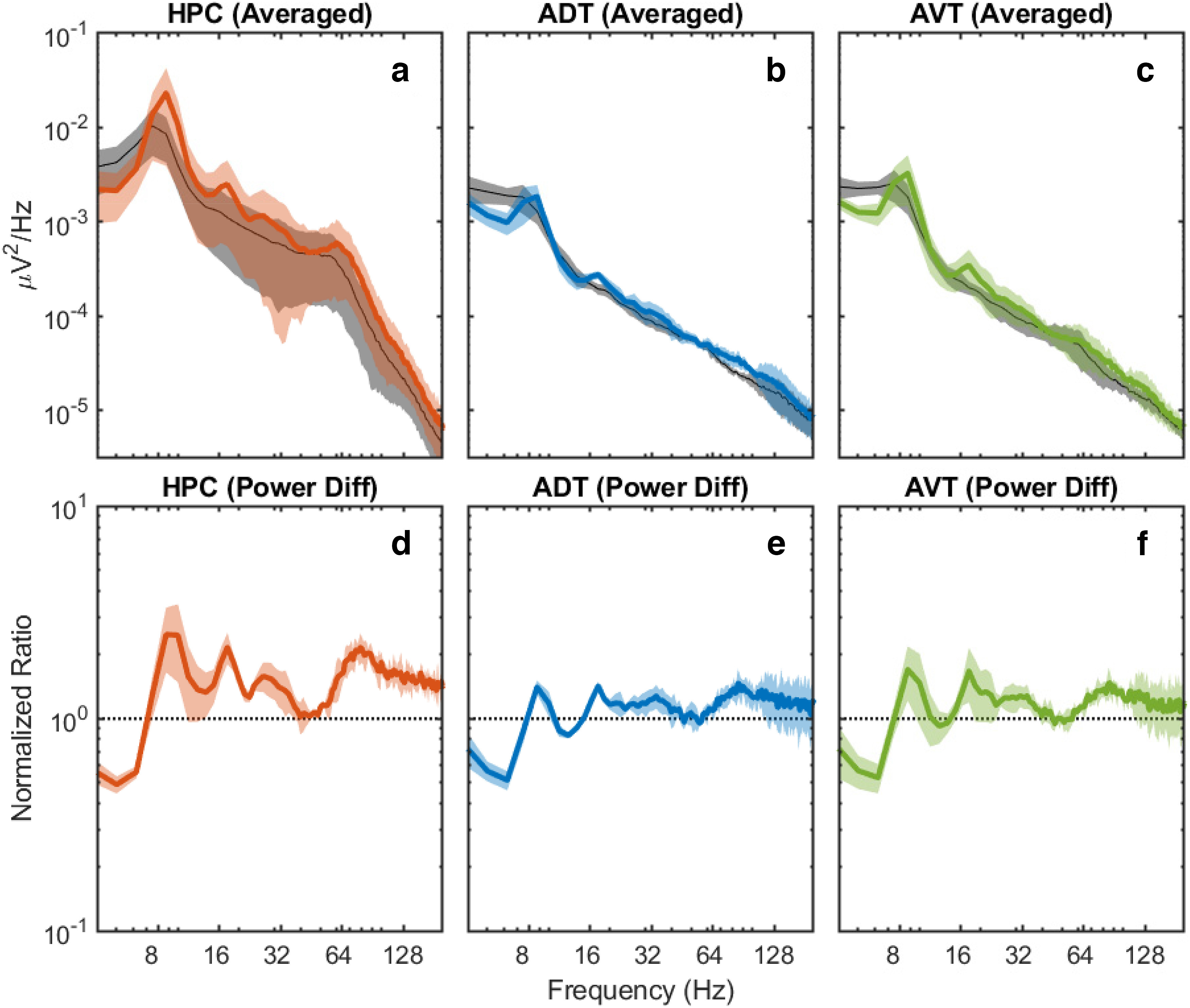
Averaged power spectral density across animals and the corresponding 95% confidence intervals for low (1–9 cm/s; black) and high (>15 cm/s; colored) speeds (***a–c***). The shaded regions are the corresponding 95% confidence intervals. The averaged power difference between high and low speeds, and the corresponding 95% confidence intervals, for hippocampal CA1, anterior dorsal thalamus and anterior ventral thalamus, respectively (***d–f***).

Similar to the observations of Ahmed and Mehta on gamma in the rat hippocampus (Ahmed and Mehta, 2012), we observed an increase in frequency and power in the gamma range of the mouse hippocampus (Fig. 2*d*). This was accompanied by increases in theta harmonics (e.g., 16, 24, and 32 Hz). Moreover, the anterior thalamus exhibited an increase in the ∼60- to 120-Hz range from low to high running speeds (Fig. 2*e*,*f*). Confidence intervals of power spectral density were calculated for low (1–9 cm/s) and high (>15 cm/s) speeds (Fig. 3*a–c*). As the absolute power may change because of individual differences and signal amplifications may also vary across animals, it may be less significant to compare, e.g., the low speed of one animal to the high speed of another animal, thus the 95% confidence interval of power difference between low and high speeds calculated for each individual animal was also provided (Fig. 3*d–f*) and showed significance. Therefore, in accord with prior results from the rodent hippocampus (Ahmed and Mehta, 2012; Zhou et al., 2019; Sheremet et al., 2020), the gamma-band power in anterior thalamus is modulated by running speed.

**Figure 4.**
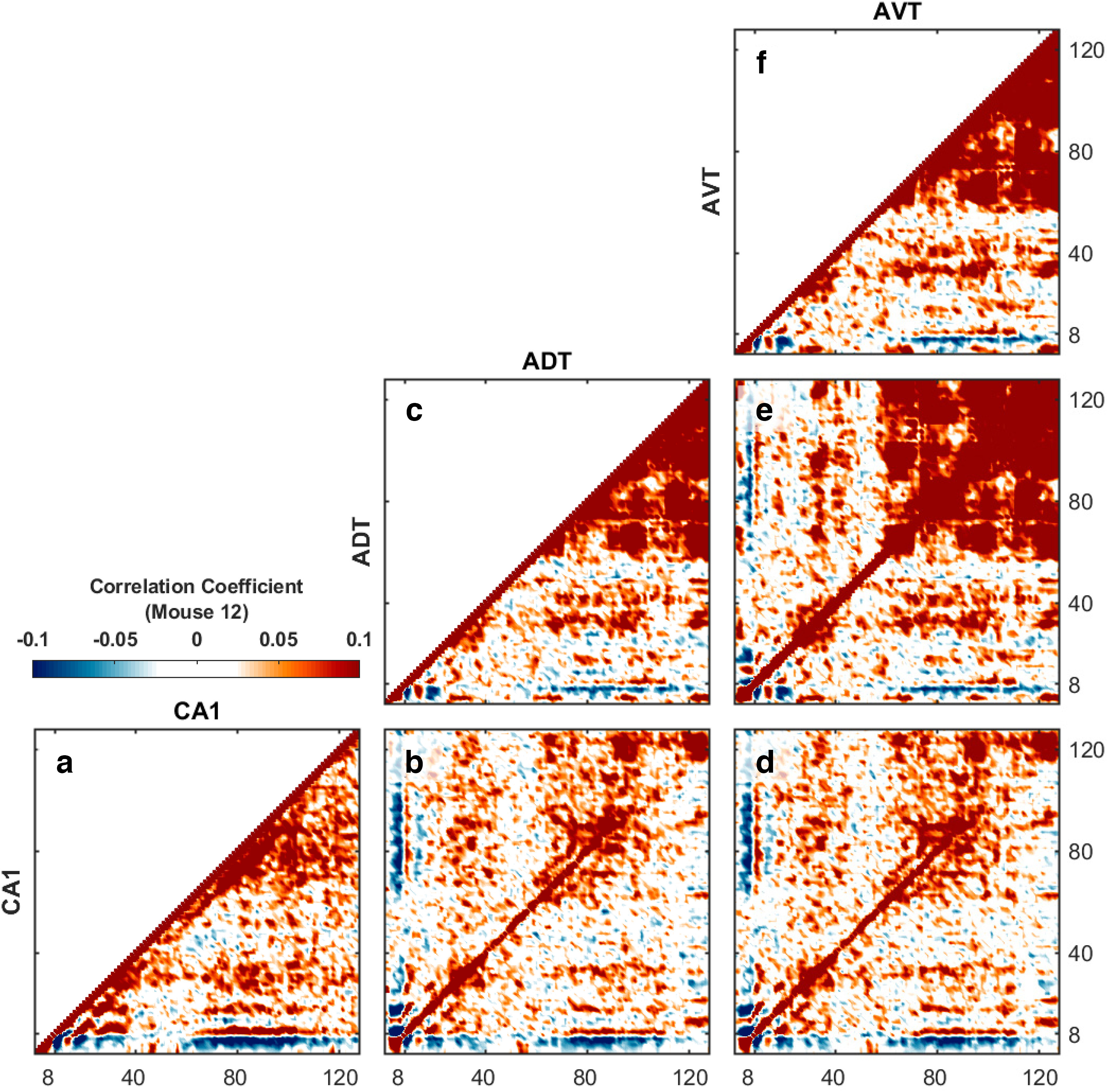
Auto-correlation and cross-correlation coefficients of Fourier transform between hippocampal CA1 and anterior thalamus (see Masimore et al., 2004, 2005). Within a region, the correlation of a frequency with itself is equal to one. As the autocorrelations are symmetric, only one-half is presented. The cross-correlation of power, however, can vary across unity and is not necessarily symmetric. Interestingly, the power of gamma in the anterior thalamus exhibits a strong correlation to both the power of theta ∼9 Hz and the first harmonic (∼18 Hz) in CA1. Gamma power in CA1, however, only exhibits a notable correlation with theta power in anterior thalamus. In all plots, correlations between frequencies less than ∼40 Hz are dominated by “dots” reflecting theta harmonics (Sheremet et al., 2016, 2019). Notably, in hippocampal CA1, there is an off-axis diagonal band ∼9 Hz away from the equal-frequency diagonal, which reflects coupling between theta power and gamma wave groups. Negative correlations are included to demonstrate that the power in some frequencies is lost as others increase (data from M12). The regional correlations are as follows: (***a***) CA1-CA1, (***b***) CA1-ADT, (***c***) ADT-ADT, (***d***) CA1-AVT, (***e***) ADT-AVT, and (***f***) AVT-AVT.

It has been proposed that auto-correlation of the power spectral density taken over time, exploring how the power in specific bands change with respect to each other, is a method of identifying unambiguous areas of frequency interaction (Masimore et al., 2004, 2005). Prior data from the mouse hippocampus has revealed co-modulation of power between theta, the first harmonic of theta, and a unitary broad gamma range (Buzsáki et al., 2003). In agreement with these results, we found off-axis correlations between 9–18 and 9–27 Hz, suggestive of theta harmonics (Fig. 4*a*). This interpretation is supported by the positive correlation of the off-axis 60- to 120-Hz band to the 9-, 18-, and 27-Hz frequencies. It should be noted that, because of the nature of harmonics, the range of frequencies covered increases, e.g., the harmonic of an 8- to 10-Hz rhythm covers 16–20 Hz, expanding the range in which cross-frequency interactions can be observed. There are prominent regions of negative correlations (e.g., 4–8 Hz). We have previously interpreted these changes as being related to the “collective enhancement of rhythmicity” (Wiener, 1965, 1966; Strogatz, 1994). Briefly, when a collection of neurons is weakly engaged, neurons with rhythmicity near 8 Hz are free to drift, with some expressing a resonance near 4 Hz and others perhaps as high as 12 Hz. With more excitatory input into the network, neurons “push and pull” on each other, effectively bringing them into common entrainment, with a loss of power in the adjacent bands.

Finally, there is a prominent diagonal band, shifted off-axis from unity in the gamma range. This is the Fourier representation of “theta modulated gamma” (Bragin et al., 1995; Chrobak et al., 2000). To illustrate, should there exist an ∼84 Hz oscillation that modulates its amplitude at 8 Hz, a decomposition into a sum of sine waves would represent this as the summation of 80 Hz and 88 Hz oscillations. Unsurprisingly, the power in these frequencies change together.

The power correlations in the anterior thalamus were similar to the hippocampus, with interactions between the 9-, 18-, and 27-Hz frequencies, indicative of harmonics, and the 9-Hz theta with a 60- to 110-Hz gamma (Fig. 4*c*,*f*). Interestingly, unlike the hippocampus, there were notable correlations between 1–3 Hz and the 25- to 40-Hz band, as well as between 1–3 Hz and the >110-Hz band. While beyond the scope of the present manuscript, we relate this to the coupling of thalamic activity to hippocampal ripples described previously (Viejo and Peyrache, 2020). Briefly, ripples tend to occur at a rate of ∼1–2 per second (Buzsáki, 2015), accounting for the Fourier power interactions between 1–3 Hz and the >110-Hz band. As ripples are discrete, nonstationary events, Fourier decomposition accounts for the polarity by ascribing a frequency that approximates the duration of the ripple. For example, the “running average voltage” of the LFP before and after a ripple, during quiescence is ∼0 mV. However, ripples are rarely symmetrical with respect to 0 mv and tend to carry a polarity that would pull the running average away from 0 mv (making the event nonstationary). This polarity should be anticipated if ripples are the biophysical consequence of a superposition of action potentials (Ylinen et al., 1995). Fourier, and other time-series analyses, will account for this nonstationary behavior as having power that is related to the duration of the event (25–40 ms; Jones, 2016). Alternatively, this band may be supported by the envelope of power associated with ripple doublets (Oliva et al., 2018). Nevertheless, as this frequency shows no power interaction with theta, it is not germane to the rest of the current presentation.

We also explored the cross-frequency power correlations between the hippocampus and anterior thalamus (Fig. 4*b*,*d*,*e*). Theta and the harmonics expressed positive power correlations across regions. Hippocampal gamma power was positively correlated to theta power in the anterior thalamus as well as the reverse; anterior thalamus gamma power was positively correlated with hippocampal theta power as well as the harmonics of theta. These data are demonstrative that there are power interactions across regions in the theta, theta harmonic, and gamma bands. Interestingly, the off-axis correlation in the gamma band between the anterior thalamus and the hippocampus takes a rectangular form in which the 60–100 Hz band in the AT exhibits high correlation to the 60- to 125-Hz range in the hippocampus, suggestive that gamma may be able to approach higher frequencies in the hippocampus, perhaps because of the higher theta power (excitatory drive) and the close proximity between interneurons and pyramidal cells in the hippocampus (Traub et al., 1996; see Discussion; Buzsáki and Draguhn, 2004). As the frequency range increases with each harmonic, it is plausible that the off-axis interaction between the anterior thalamus 27- to 36-Hz power and hippocampal gamma reflects overlapping bins of theta harmonics or is related to a novel band.

**Figure 5.**
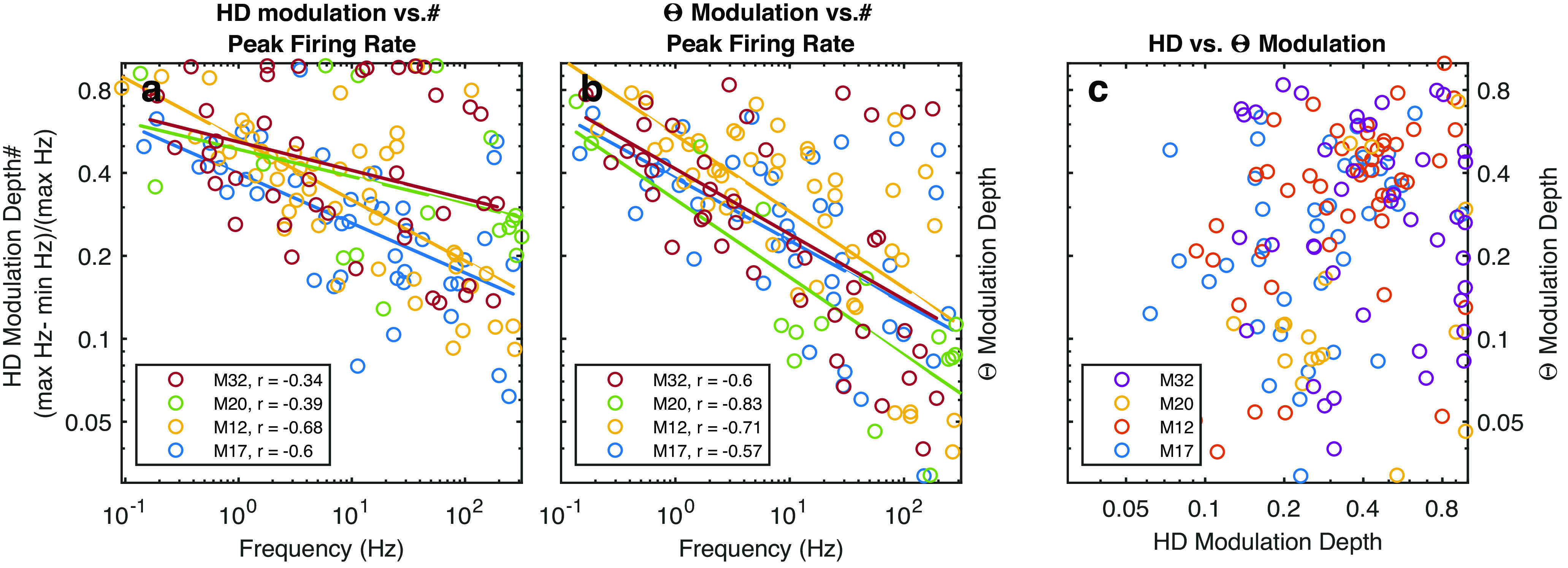
The relationship between neuron firing rate, theta phase modulation and head direction across mice. ***a***, Scatter plot of HD modulation depth versus cellular peak firing rate for all mice. The HD modulation depth are defined as (max Hz − min Hz)/(max Hz) of the “directional” firing rates. In the figure, each dot represents an individual cell, and different mice are denoted by different colors. The solid lines are linear regression fit of the values for individual mice using the same color code. Note that the negative correlation between modulation depth and firing rate, confirmed by the Pearson *r* values, for individual mice r values are −0.34, −0.39, −0.68, −0.60. ***b***, Scatter plot of theta phase modulation depth versus cellular peak firing rate for all mice. Note that the negative correlation between modulation depth and firing rate are confirmed by the Pearson *r* values, for individual mice r values are −0.6, −0.83, −0.71, −0.57. ***c***, Scatter plot of HD modulation versus theta phase modulation. Note that there is not an obvious correlation between the modulations.

**Figure 6.**
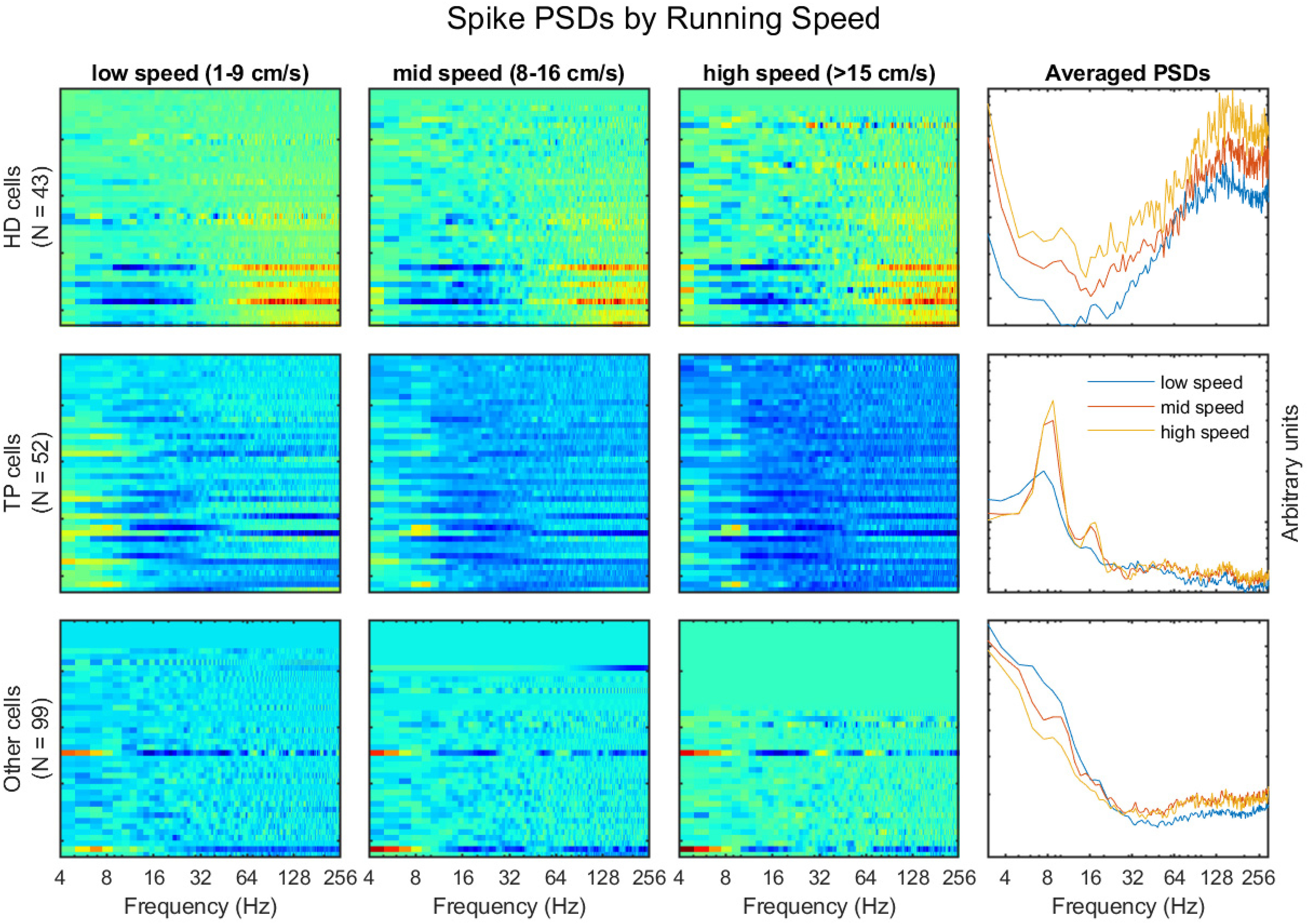
Power spectral density of neuron spike trains as a function of velocity. Left panels, Individual power spectra for spike trains at low running velocity (1–9 cm/s) sort by cell types [(1) HD cells; (2) theta modulated cells; (3) other cells]. Note that each cell was normalized by its mean power, resulting in power being presented in arbitrary units and the color axis is logarithmic. Rows of each plot are sorted by the mean firing rate of individual cells. Mid panels, Same as left panels but for mid (8–16 cm/s) and high running speeds (>15 cm/s). Right panels, Averaged power spectra of the cell populations of the three types at low, mid, and high velocities. Note that because of the binary nature of the spike traces, a burst of spikes will have a positive mean within the time series, giving an odd presentation of low-frequency power. Thus, the frequency range for these plots started at 4 Hz.

To investigate the interaction between spike modulation and head-direction, neuron firing rate, theta phase modulation, and head direction were correlated across mice (Fig. 5). There was a strong inverse correlation between firing rate and head direction selectivity, with the highest firing rate neurons having the least amount of modulation to heading. There was also a strong correlation between firing rate and theta modulation, with the highest firing rate neurons having the least modulation by theta phase. Using these data, neurons were classified as neurons with directional selectivity (HD cells), neurons with theta phase selectivity (theta phase cells) other cells. The spike-frequency modulation was studied by calculating the power spectral density of spike trains as a function of velocity (Fig. 6). Individual power spectral densities of each cell were sorted by their firing rates. As the firing rate can alter the representation of normalized before averaging, each neuron’s firing rate was normalized before averaging. Similar to Figure 1, the overall power spectral density developed a prominent peak in the theta range as running speed increased, majorly contributed by the theta phase modulated neurons. There was also a prominent peak at ∼16 Hz of the non-HD, theta phase modulated cells suggesting theta harmonic modulation, which is less notable in the HD cells. At higher frequencies, the spike train power spectral density of HD cells shows a significant bump over ∼60 Hz, while the spike train power spectral density of non-HD cells was mostly unstructured although there was a mild but notable increase around theta band with velocity and perhaps some broadband gamma modulation.

It has previously been reported that hippocampal neurons are modulated by both theta and gamma simultaneously (Bragin et al., 1995; Zhou et al., 2019). As action potentials are quantal events, a parsimonious explanation is that neurons fire in gamma burst, with an interburst interval that matches theta (Lisman and Idiart, 1995), rather than an alternative model in which the neurons that support theta rhythmicity are orthogonal to those that support gamma. This suggests that oscillations are not independent but rather interdependent. Therefore, to demonstrate the presence of harmonics and investigate the cross-frequency coupling, we conducted bicoherence and cross-bicoherence analyses.

**Figure 7.**
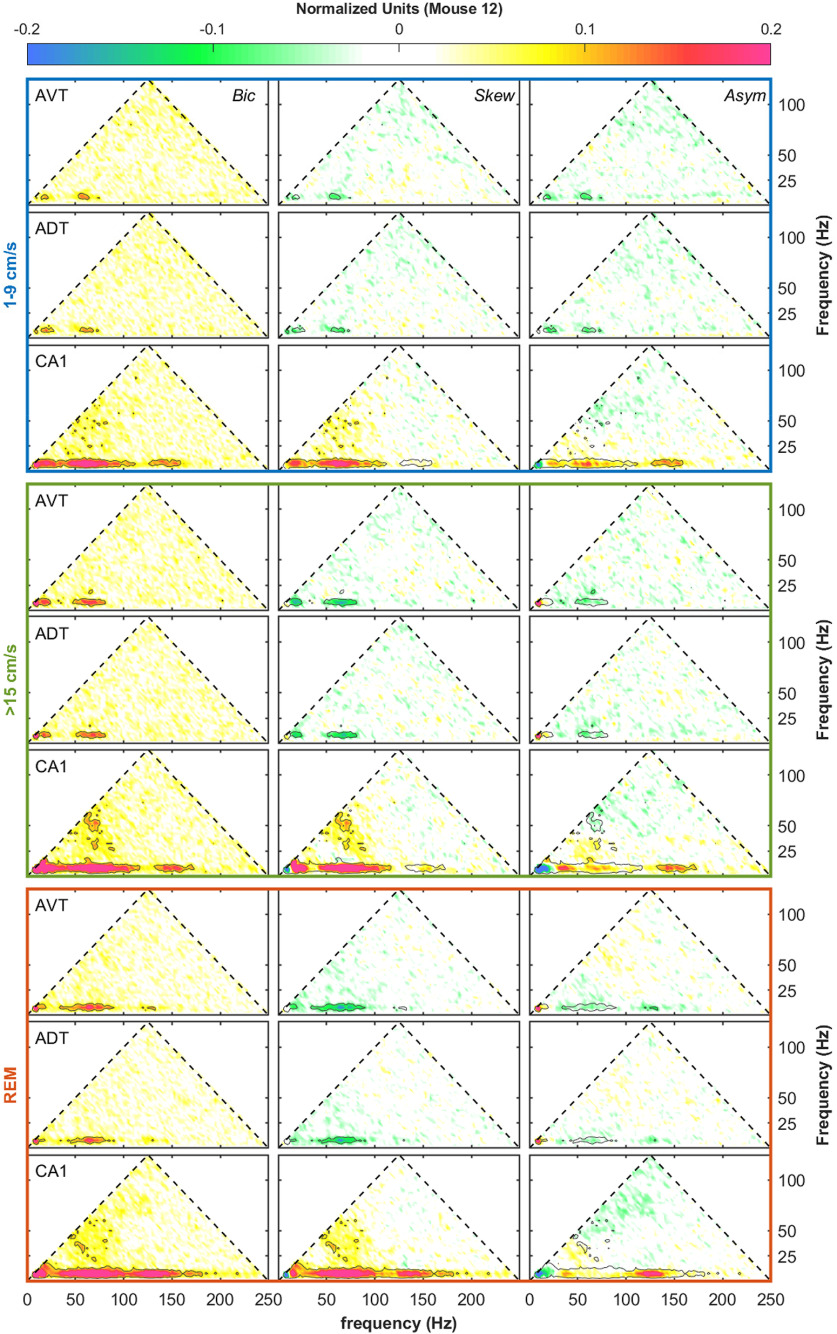
Dependency on mouse speed of bispectrum of LFP in hippocampal CA1 and anterior thalamus subregions. Left to right, The absolute value of the bispectrum, real (related to LFP skewness) and imaginary (related to LFP asymmetry) part of the bispectrum. Top to bottom, Low speed (1–9 cm/s), high speed (>15 cm/s), and REM for each region in AVT, ADT, and CA1, respectively. An in-depth explanation of the bicoherence plot can be found in previously published work (Sheremet et al., 2016). Both theta-theta harmonic and theta-gamma phase coupling show an increment with increased speed. The CA1 region also exhibits some gamma-gamma coupling indicative of skewed gamma oscillations. Overall, the CA1 exhibits stronger interactions than the thalamus. Theta-gamma band in the thalamus and CA1 show opposite skewness, implying the interactions are maximum at different phases of theta (data from M12).

We have previously used bispectral analysis to demonstrate an increase of theta, theta harmonic, and gamma cross-frequency interactions as a function of running speed in the rat hippocampus (Sheremet et al., 2016, 2019, 2020). In the present study, we observed a well-ordered increase of nonlinear coupling as a function of speed in both the CA1 region of the hippocampus and the anterior thalamus (Fig. 7). However, the strength of cross-frequency coupling in the anterior thalamus was relatively low at low running speeds relative to the CA1 region. Within the 1–9 cm/s speed bin, the CA1 region exhibited coupling up to the 27-Hz harmonic of theta as well as theta gamma coupling that exceeded what was seen in the anterior thalamus. It is worth emphasizing that the development of theta harmonics strongly coupled to theta indicates a nonlinear deformation of the theta rhythm, which takes the form of wave skewness (“sawtooth”), asymmetry (e.g., a cnoidal shape that is also symmetric on either side of the peak), or both (Sheremet et al., 2016). The respective contribution of skew and asymmetry, features of the bicoherence, increase as a function of velocity (Sheremet et al., 2016, 2018b). During REM, the magnitude of theta-theta harmonics coupling is in between low and high velocity, while there is a noticeable stretch of bandwidth in high frequency comparing to high speed, possibly because of the presence of ripples.

**Figure 8.**
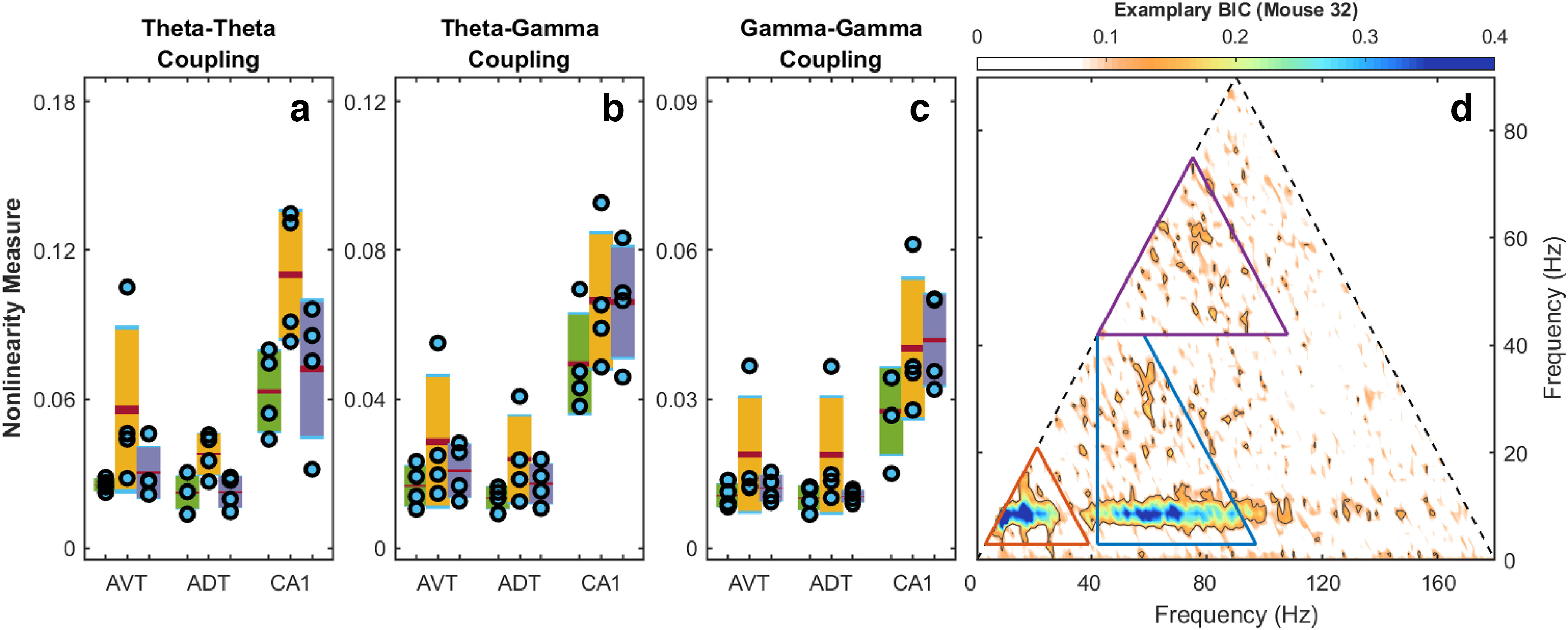
Estimate of nonlinear cross-frequency phase coupling. ***a***, The overall strength of theta-theta harmonics nonlinearity was estimated by summing the bicoherence values in the red triangle in the panel at the right. Estimates are shown for speed bins at 1–9 cm/s (green), >15 cm/s (yellow), and REM (purple). ***b***, The strength of nonlinear coupling between theta (and harmonics) and gamma rhythms was estimated by summing the bicoherence values in the blue trapezoid in the panel on the right. ***c***, The strength of gamma-gamma coupling. Hippocampal CA1 exhibits a significantly larger coupling strength compared with anterior thalamic regions. Both theta-theta harmonics and theta-gamma coupling strength show significant variability as a function of mouse speed. Note that the *y*-axis differs between nonlinearity measures to optimize for the display of the theta-gamma nonlinearity. The red line is the mean, green, yellow or purple is 1.90 of the SE, and light blue denotes the boundaries of 1 SD. ***d***, Regions used for estimating the strength of theta-theta harmonics (reg triangle) and theta-gamma coupling (blue trapezoid) are bounded by frequency intervals [4, 40 Hz] for theta and [42, 100 Hz] for gamma.

Because the location and magnitude of the bicoherence peaks identify and measure the intensity of cross-phase interactions, the strength of the nonlinear coupling can be quantified by integrating the bicoherence over the region of interest. Therefore, we examined theta-theta harmonic nonlinear coupling and theta-gamma nonlinear coupling in the hippocampal CA1 region and the anterior thalamus as a function of running speed (Fig. 8). A repeated measures two-factor ANOVA was run for each region of interest separately with Tukey’s *post hoc* multiple comparisons tests. The repeated measures were nonlinearity (theta-theta, theta-gamma, and gamma-gamma), and behavioral state [low running speed (1–9 cm/s), high running speed (>15 cm/s), and REM]. For the antero-ventral thalamus, there was a main effect of nonlinearity (*F*_(1,2)_ = 60.36, *p* = 0.0022) and behavioral state (*F*_(1,2)_ = 19.17, *p* = 0.0215), but the interaction did not reach statistical significance (*F*_(1,2)_ = 2.857, *p* = 0.1760). The Tukey’s multiple comparisons test indicated that the mean value of theta-gamma nonlinearity was lower at low running speed compared with high running speed (*p* = 0.0033, 95% CI = [0.006899, 0.003105]), and gamma-gamma nonlinearity was lower at low running speed compared with REM (*p* = 0.0237, 95% CI = [0.002346, −00003251]). For the antero-dorsal thalamus, there was a main effect of nonlinearity (*F*_(1,2)_ = 14.63, *p* = 0.0134), but not behavioral state (*F*_(1,2)_ = 4.365 *p* = 0.1263). Additionally, there was not a statistically significant interaction effect (*F*_(1,2)_ = 2.262, *p* = 0.1767). In the CA1, there was a main effect of nonlinearity (*F*_(1,4)_ = 12.46, *p* = 0.0218) and behavioral state (*F*_(1,4)_ = 24.42, *p* = 0.0015), as well as an interaction effect between the two (*F*_(1,4)_ = 13.13, *p* = 0.0050). Tukey’s test for multiple comparisons found that the mean value of theta-theta nonlinearity was lower at both low running speed (*p* = 0.0068, 95% CI = [0.06979, 0.02417]) and during REM (*p* = 0.0336, 95% CI = [0.005299, 0.07048]) compared with high running speed. The mean value of theta-gamma nonlinearity was lower for low running speed compared with both high running speed (*p* = 0.0316, 95% CI = [0.03115, 0.002695]) and REM (*p* = 0.0382, 95% CI = [0.03153, 0.001618]). Finally, gamma-gamma nonlinearity was lower for low running speed compared with REM (*p* = 0.0089, 95% CI = [0.02204, 0.006679]). Interestingly, although the anterior thalamic regions had trends to increase nonlinear coupling with velocity, the effects were not statistically significant. This may be partially accounted for by the overall lower power across all frequencies in the anterior thalamus relative to the CA1 region (Fig. 2). When nonlinearity in CA1 versus the anterior thalamic nuclei were directly compared, there was a significant effect (*F*_(2,27)_ = 39.64, *p* < 0.001). *Post hoc* analysis indicated that nonlinearity was greater in CA1 compared with both AVT and ADT (*p* < 0.001, 95% CI = [0.025–0.050] for both comparisons). Nevertheless, the non-zero power-power (Fig. 4) and phase-phase interactions (Fig. 7) in the anterior thalamus suggests that this region may be influenced by hippocampal input but have attenuated parametric space to change as a function of velocity. To explore this idea, we therefore examined frequency interactions across regions using cross-bicoherence.

**Figure 9.**
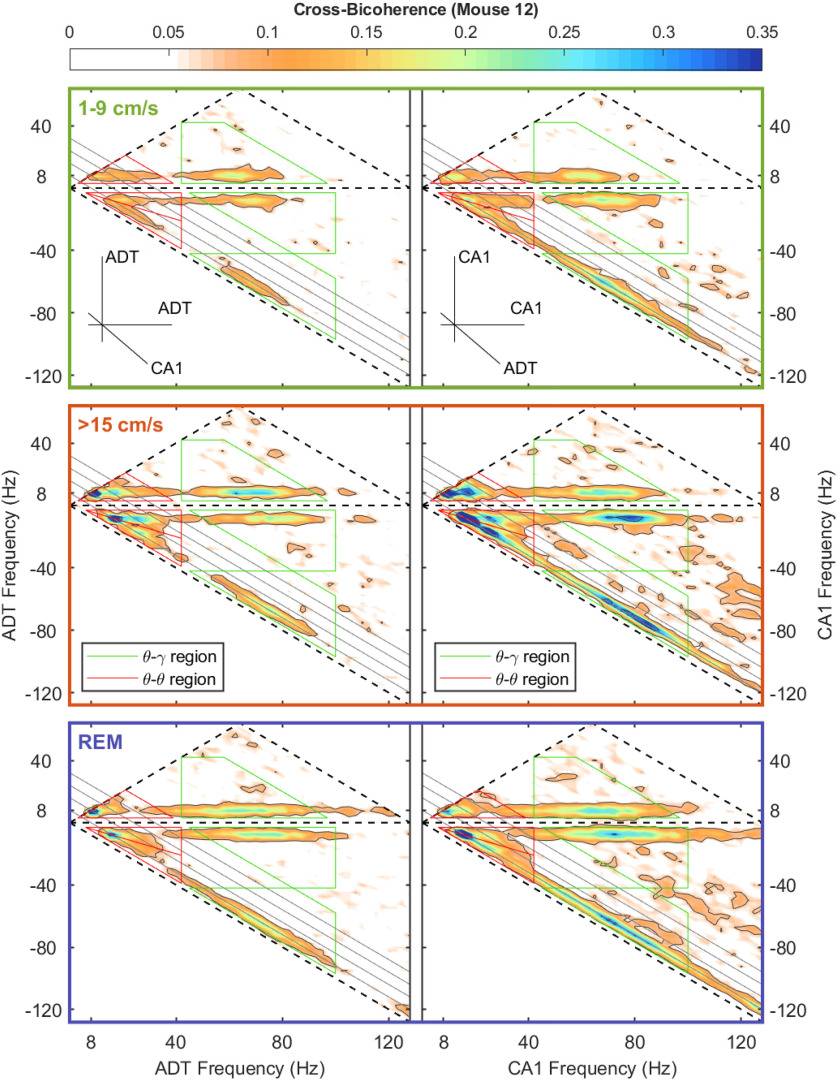
Dependency of mouse speed of ADT-hippocampal CA1 cross-bicoherence of the type $B_{mn}^{TTH}$ (left) and $B_{mn}^{HHT}$ (right). Top, The absolute value of the cross-bispectrum at low speed (1–9 cm/s). Mid, The absolute value of the cross-bispectrum at high speed (>15 cm/s). Bottom, The absolute value of the cross-bispectrum during REM. Frequency domains relevant for cross-region triads of the type $\left({\text{θ}}^{T},{\text{θ}}^{T},{\text{θ}}^{H}\right)$ and $\left({\text{θ}}^{T,H},{\text{γ}}^{H,T},{\text{γ}}^{T}\right)$ are circumscribed by red triangles and blue trapezoids, respectively, in the left panels; frequency domains relevant for cross-region triads of the type $\left(\theta^{H},\theta^{H},\theta^{T}\right)$ and $\left({\text{θ}}^{H,T},{\text{γ}}^{T,H},{\text{γ}}^{H}\right)$ are circumscribed by blue triangles and purple trapezoids, respectively, in the right panels. Domains used for circumscribing theta-theta harmonics (blue triangles) and theta-gamma coupling (purple trapezoids) are bounded by frequency intervals [4, 40 Hz] for theta and [42, 100 Hz] for gamma (data from M12).

**Figure 10.**
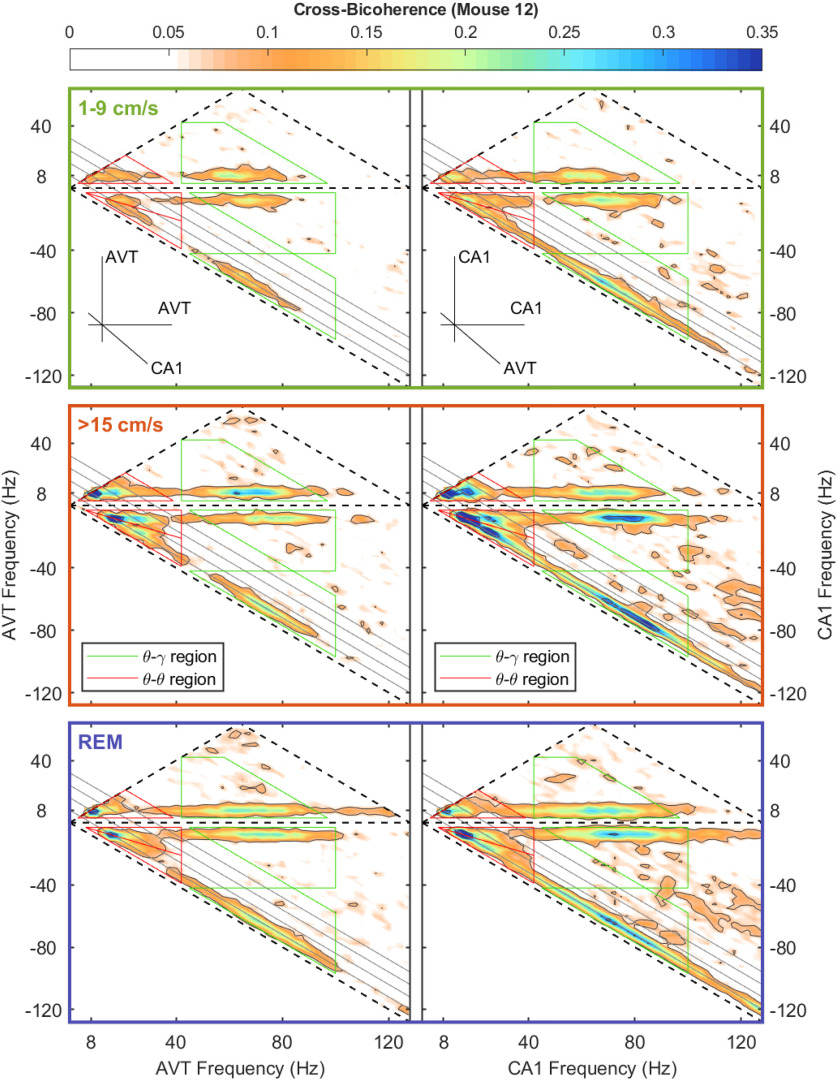
Same as Figure 8 but for dependency of mouse speed of AVT-hippocampal CA1 cross-bicoherence.

**Figure 11.**
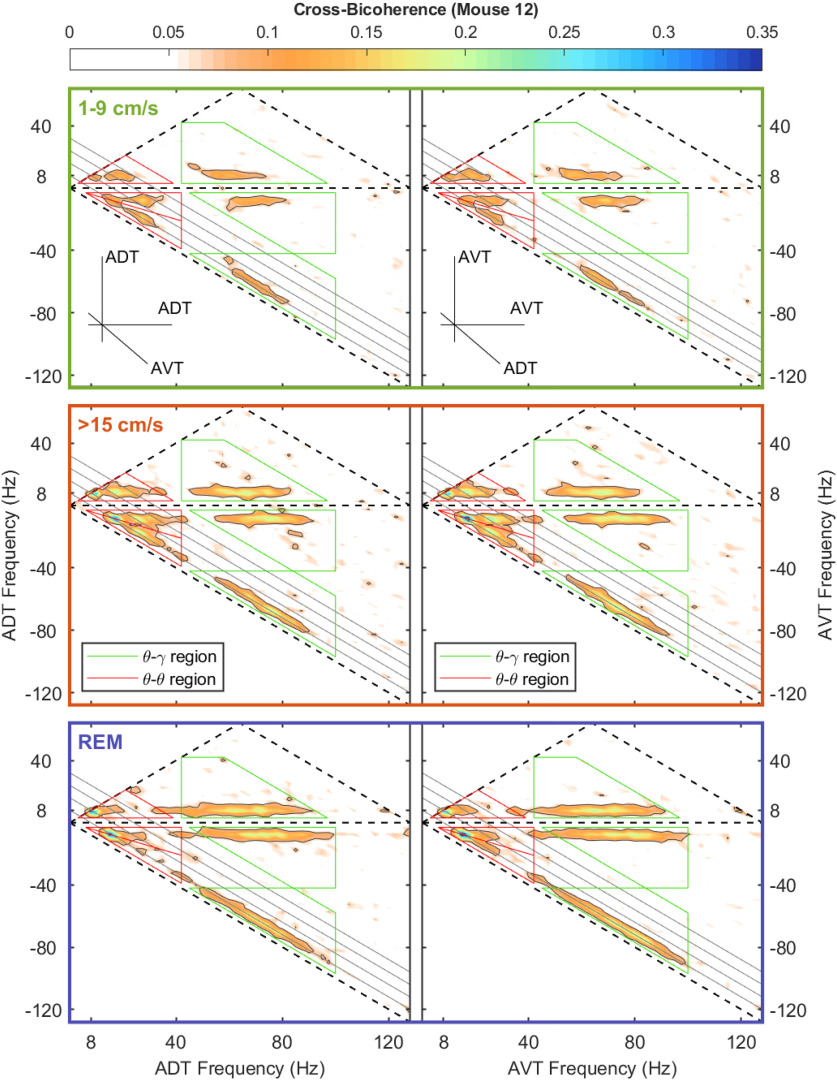
Same as Figure 8 but for dependency of mouse speed of AVT-ADT cross-bicoherence.

Similar to bicoherence, cross-bicoherence measures a nonlinear three-way phase coupling with the added extension of looking across brain regions. As the current study is interested in theta-theta and theta-gamma interactions across the anterior thalamus and CA1 region of the hippocampus and cross-bicoherence is a three-wave interaction, there are a multitude of ways that oscillations can interact. For instance, in terms of theta-theta harmonic coupling across regions can take the form of (1) 8–16–24 Hz (CA1–CA1–AT), (2) 24–8–16 Hz (CA1–CA1–AT), (3) 24–16–8 Hz (CA1–CA1–AT), and so on, where these intersections represent a triad phase correlation between integer frequencies of theta (Fourier modes with frequencies *f*_n_, *f*_m_, and *f*_n+m_ = *f*_n_ + *f*_m_). Considering this, there are three regions of the cross-bicoherence plot that describe theta-theta harmonic interactions across regions (red triangles; Figs. 9-11). Similarly, there are also three regions that describe cross-regional theta-gamma oscillations (green trapezoids), where the initial phase of two oscillations can either add or subtract to equal the phase of the third oscillation. For example, in the instance of “theta modulated gamma,” the difference in the initial phase between an 88- and an 80-Hz oscillation would equal the phase of an 8-Hz rhythm. Moving forward, we will use the nomenclature of appending the name of the frequency band with a superscript that corresponds to the brain region; for example, θ^H^θ^H^θ^T^ will be used to denote a cross-bispectral term that involves the three components in the domain of theta and harmonics, with the first two components in the CA1 region of the hippocampus (H) and the third in the anterior thalamus (T). We refer the reader to our prior work for further information on how to interpret these plots (Sheremet et al., 2020). Examining the cross-bicoherence plots as a function of velocity, qualitatively all forms of theta-theta and theta-gamma coupling increase between the CA1 region and anterior thalamus as a function of animal running speed.

**Figure 12.**
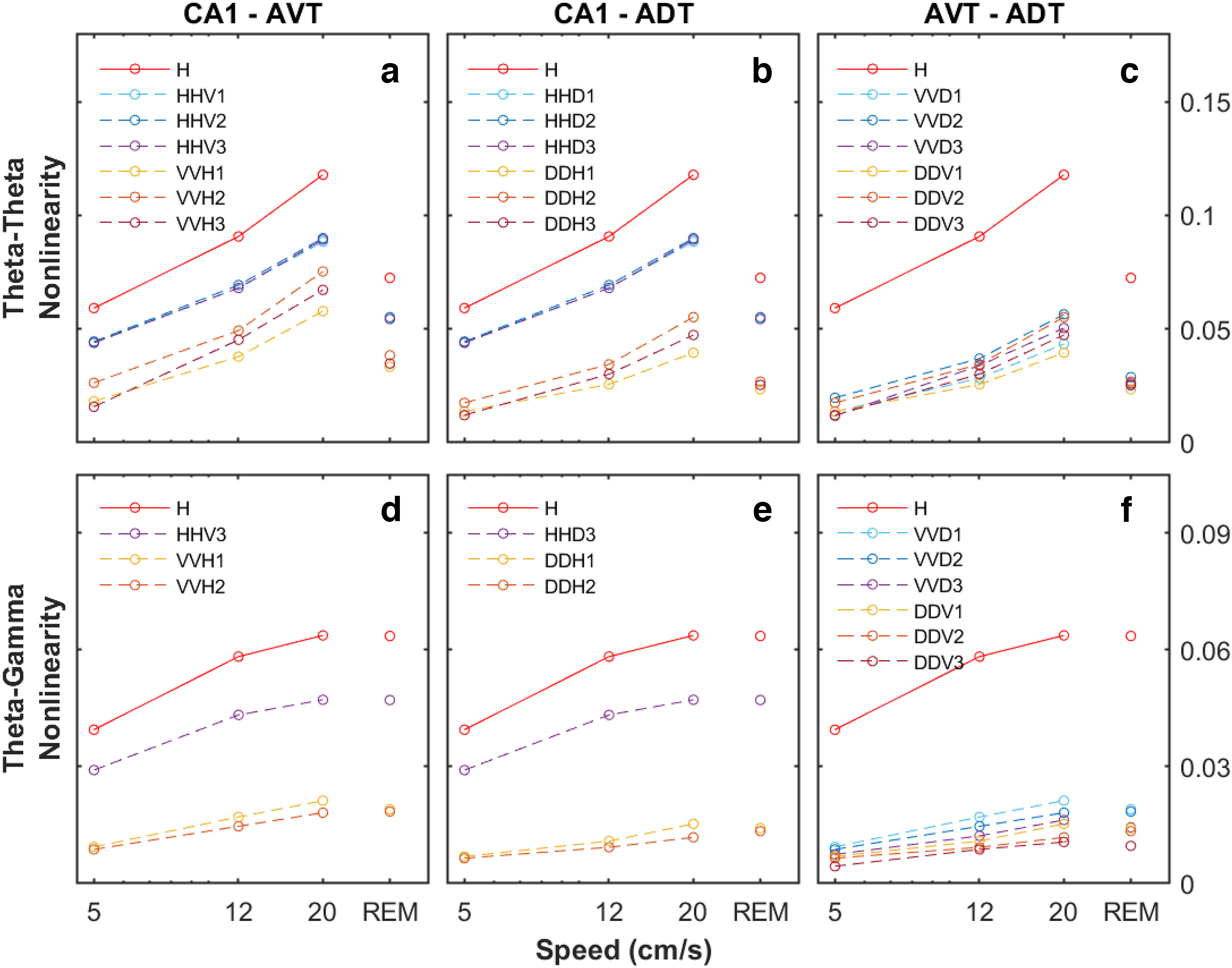
Averaged magnitude of nonlinear theta-gamma and theta-theta harmonics cross-region coupling of hippocampal CA1 versus AV thalamus, hippocampal CA1 versus AD thalamus, and AV thalamus versus AD thalamus, compared with same-region coupling within hippocampal CA1 as a function of mouse speed. Top panels, Magnitude of thalamic-hippocampal cross-region theta-theta harmonics coupling of the types $\left(\theta^{1},\theta^{1},\theta^{2}\right)$ and $\left(\theta^{2},\theta^{2},\theta^{1}\right)$ compared with the magnitude of hippocampal-hippocampal coupling (***a***, CA1-AVT theta-theta coupling; ***b***, CA1-ADT theta-theta coupling; ***c***, AVT-ADT theta-theta coupling); ; Bottom panels, Magnitude of thalamic-hippocampal cross-region theta-gamma coupling of the types $\left({\text{θ}}^{1},{\text{γ}}^{1},{\text{γ}}^{2}\right)$ and $\left({\text{θ}}^{2},{\text{γ}}^{2},{\text{γ}}^{1}\right)$ compared with the magnitude of hippocampal-hippocampal coupling (***d***, CA1-AVT theta-gamma coupling; ***e***, CA1-ADT theta-gamma coupling; ***f***, AVT-ADT theta-gamma coupling).

The quantitative breakdown of these relationships for each mouse is presented in Figure 12. Each subplot depicts either the theta-theta or theta-gamma aggregate nonlinearity within each triangle or polygon across running speed. For instance, as there are three ways in which θ^1^θ^1^θ^2^ (where superscripts index regions) can interact and three ways that θ^2^θ^2^θ^1^ can interact, there are a total of six different measures of theta-theta harmonic interactions. The CA1-CA1 regional interaction is shown for comparison. Across all mice, the rates of theta-theta harmonic nonlinear coupling increased at similar rates with increasing running speed. With respect to theta-gamma interactions, there are total 6 regions of theta-gamma per animal per region pair (two groups of three, using either anterior thalamus theta or CA1 theta): (1) θ^1^ γ^1^ γ^2^, θ^1^ γ^2^ γ^1^, θ^2^ γ^1^ γ^1^ and (2) θ^2^ γ^2^ γ^1^, θ^2^ γ^1^ γ^2^, θ^1^ γ^2^ γ^2^ (for further information, please see Sheremet et al., 2020, their Figs. 7 and 8). Again, a consistent pattern emerges in which nonlinear coupling increases with running speed. The largest magnitude of coupling involves the θ^H^ γ^H^ γ^T^ frequency triad, followed by a modest increase in the nonlinear coupling of interactions of the θ^T^ γ^T^ γ^H^.

## Discussion

### Summary

In the current manuscript, we analyzed mouse data from simultaneous anterior thalamus and CA1 hippocampal recordings generously provided by Dr. Buzsáki, Dr. Peyrache, and colleagues (Peyrache et al., 2015a). While cross-frequency theta-gamma coupling within the rodent hippocampus has been described extensively (Montgomery et al., 2008; Schomburg et al., 2014; Scheffer-Teixeira and Tort, 2016; Fernández-Ruiz et al., 2017; Trimper et al., 2017; Amemiya and Redish, 2018), the local-field potential in the thalamus has received comparatively less attention. As the anterior thalamus receives input from the entorhinal cortex, subiculum, and the parasubiculum and postsubiculum (Seki and Zyo, 1984; van Groen and Wyss, 1990a, b; Wright et al., 2010), and projects back to the entorhinal cortex, presubiculum, parasubiculum, and postsubiculum (Wyss et al., 1979; Amaral and Cowan, 1980; Shibata, 1993; Shibata and Kato, 1993; van Groen and Wyss, 1995; van Groen et al., 1999; Jankowski et al., 2013), it stands to reason the interaction between the hippocampal areas and the anterior thalamus take the form of reentrant volleys of synaptic activity (for a comprehensive review of the anatomy, please see Jankowski et al., 2013; McNaughton and Vann, 2022). While the ADT and AVT receive projections from the lateral mammillary bodies and medial mammillary bodies, respectively (Watanabe and Kawana, 1980; Shibata, 1992), potentially offering a division between head direction and theta mechanisms, the medial and lateral mammillary nuclei receive common inputs from the septum and supramammillary nucleus (Gonzalo-Ruiz et al., 1992) suggestive of a common orchestrating mechanism. Phasic reentrant connectivity between two (or more) regions comprises a simple mechanism by which regions can rhythmically engage each other (Edelman, 1987; Buzsáki and Draguhn, 2004). Therefore, as hippocampal theta power increases with running velocity (McFarland et al., 1975; Hinman et al., 2011; Richard et al., 2013; Dannenberg et al., 2020), we were interested in how the LFP in the anterior thalamus reorganized as a function of running speed.

Complementing research from the rat hippocampus, we found that there is a redistribution of power in the mouse hippocampal power spectra with a decrease in low frequencies (∼1–4 Hz) and an increase in theta, theta harmonic, and gamma power with increases in running speed. Relative to the hippocampus, the power spectral density of the anterior dorsal and anterior ventral thalamus LFP was substantially lower, although power in the theta, theta harmonic, and gamma band ranges also increased with running speed (Figs. 2, 3). The cross-correlation in power spectral densities revealed that power changes in these bands were also positively correlated with each other, such that as theta power increased, so did the activity in the gamma band (Fig. 4). Interestingly, in the mouse CA1 region, an off-axis diagonal band, parallel with the unity line but offset by ∼8 Hz, was prominent (Fig. 4*a*,*b*,*d*). We interpret this off-axis diagonal to be the spectral expression of “theta-modulated gamma” where, should the amplitude of gamma wax and wane in amplitude at 8 Hz, the Fourier decomposition into sine waves represents this as an interference pattern between two oscillations (e.g., 80- and 88-Hz sine waves). Examining the regions of theta-gamma coupling, the power-power and bicoherence analyses were suggestive that gamma did not extend as high in the anterior thalamus relative to the hippocampus. From the opposite perspective, the hippocampus can support a higher power, higher frequency gamma rhythm relative to the anterior thalamus, resulting in the rectangular gamma correlation in the cross-regional power spectral cross-correlations (Fig. 4).

By looking at modulation depths of anterior thalamic neurons as a function of peak firing rate, we found that the lower peak firing neurons expressed either higher directional selectivity or higher theta modulation (Fig. 5). Based on the sorting of neurons into either head direction or theta modulated neurons (Figs. 1, 5), spike trains were analyzed via Fourier analysis (Leung and Buzsáki, 1983). Unsurprisingly, theta preferring neurons exhibited theta and the 16-Hz harmonic in their spike trains, which became more prominent as running speed increased (Fig. 6). In comparison, head direction cells exhibited some modulation to theta which increased with velocity. However, this modulation was eclipsed by power in the low (>4 Hz) and high (<100 Hz). The modulation by low frequency can be explained behaviorally as a significant amount of time passing between a mouse experiencing the same head direction consecutively (the mouse may run north and then turn east; seconds may pass before facing north again). The high frequency modulation on the other hand may be related to bursting in a subpopulation of neurons, the spectral representation of the near instantaneous firing rate of neurons firing in succession (e.g., a 10-ms interspike), or a mixture of both (Stackman and Taube, 1997). Neurons with little to no directional selectivity or theta modulation expressed some spike train modulation to theta and the gamma band that became more prominent with higher running speeds (Fig. 6).

The bicoherence analysis on the LFP revealed that similar to the rat (Sheremet et al., 2019), there was an increase in theta-theta harmonic and theta-gamma nonlinear coupling as a function of running speed in the mouse CA1 region. While there was a trend for the anterior thalamus to follow a similar pattern in the nonlinear coupling, it did not reach statistical significance (see below, Limitations; Figs. 7, 8). With the lower amount of power in the anterior thalamus, there is an associated decrease in the variance within a band (i.e., the amplitude range of an 80-Hz anterior thalamus rhythm is approximately one order of magnitude than the range that an 80-Hz rhythm can vary in amplitude in the hippocampus). This decreased parametric space suggests that nonlinear cross-frequency coupling within the anterior thalamus moves from “weak” to “slightly less weak” as velocity increases. Nevertheless, as there were power-power and phase-phase interactions, we considered that the anterior thalamus may be influenced by hippocampal input. Therefore, we ran cross-bicoherence analyses to determine cross-regional, cross-frequency coupling. Interestingly, across all animals, there was an increase in theta-theta (and harmonic) and theta-gamma nonlinear cross-regional coupling with running speed (Figs. 8-11). The theta-theta cross-regional nonlinear coupling was greater than the theta-gamma nonlinear coupling. While at first glance, it may seem obscure to discuss three-way cross-regional coupling, a notation such as $\left({\text{θ}}^{T},{\text{γ}}^{H},{\text{γ}}^{H}\right)$ follows from the “theta-modulated gamma” as described above, where the envelope of the gamma rhythm in the anterior thalamus is coupled to hippocampal theta. Parsimoniously, however, it can be considered that both theta-theta and theta-gamma cross frequency coupling increases across all regions as a function of increasing running speed (Fig. 12).

### Limitations

Before placing the current results into a broader context, it is prudent to describe the limitations of the study. First, there are favorable and unfavorable consequences when working from a shared database. On a positive note, there is the potential to provide an expansive story, especially when multiple brain regions are recorded simultaneously. Furthermore, it provides research to be conducted in a manner that respects animal welfare, reducing the total number of animals needed to enhance our understanding. However, a downside of this is that, while the approach and animal number were optimized by the initial researchers, our study may have benefited from implementing other behavioral tests (e.g., mnemonic tests), more animals, or different electrode configurations. For instance, there was a nonsignificant trend for nonlinearity to increase in the anterior thalamus. This may become significant should additional animals be added to the analysis. However, this action in itself becomes an exercise in significance chasing by seeking the “N” that will confirm an a priori idea (Szucs, 2016), and may more generally speaks to the shortcomings of traditional null hypothesis significance testing (Krantz, 1999; Branch, 2014; Szucs and Ioannidis, 2017). Therefore, we leave the “trend” in bicoherence coupling for the interpretation of the reader. However, we consider the results to be in favor of the idea that nonlinear cross-frequency coupling does increase in the thalamus as a function of velocity, although the magnitude of the phenomenon is relatively small. A second limitation is that we have used the terms “rhythms” and “oscillations” not in their original physical meanings. While this has relevance in terms of how the Fourier decomposition represents power and phase, it is not to imply that the nervous system operates as a literal pendulum or clock or that the nervous system has evolved to generate pure sinusoidal oscillations.

### Broader implications

On the contrary, the nervous system evolved against the selective pressure of rapid environmental changes (i.e., “anticipate” what happens next and respond appropriately). A simple architecture that could support this would be an “input-output” reflex (however, reflexes tend to be rigid, offering little space for learning or adaptation). Research from the stomatogastric ganglion demonstrates that a small network can express multiple stable patterns depending on physiological conditions (Weimann et al., 1991), demonstrating that the network can be rapid, robust (able to recover patterns), and adaptable, forming the appropriate spatiotemporal pattern to match the environment. Scaling up, evolution favored an anatomically fractal architecture with small recurrent loops nested within larger loops (Edelman, 1987; Buzsáki et al., 2004; Sporns, 2006; Seth and Edelman, 2009). It was this recurrent connectivity traced by Lorente de Nó (1938) that inspired Donald Hebb to postulate that dynamic spatiotemporal patterns across a population of neurons, “cell assemblies” and “phase sequences,” are the functional unit that organizes behavior (Hebb, 1949; Nadel and Maurer, 2020; Brown, 2020; Maurer and Nadel, 2021). Activity propagating through the nervous system, producing these dynamic spatial-temporal patterns, can be measured on the microscale level as the action potentials of individual neurons or on the mesoscale, as the LFP. Simply stated, LFP is primarily the synaptic reflection of spatiotemporal pattern formation among the units. As rapid spatiotemporal pattern formation is the neurophysiological dynamic across a network of nested, reentrant loops that supports survival, it should come as no surprise that rhythmic activity is well conserved across mammalian brains (Buzsáki et al., 2013). While it may be tempting to relate rhythms to synchrony, suggestive of a repetitive, static pattern of activity, a comprehensive account should discuss the local-field potential reflecting the reverberatory dynamic progression of activity across networks.

Along these lines, it needs to be stated that the power in the 8-Hz band during rest or quiescent states, when theta is not readily observable by the eye, remains ∼2–3 orders of magnitude above the true electrothermal background (Zhou et al., 2021). As the LFP is primarily carried by synaptic transmembrane current (Buzsáki et al., 2012), this magnitude of low-frequency power in quintessential “non-theta states” suggests the opposite- the circuits of the nervous system that are responsible for theta are always active, propagating barrages of synaptic activity between 7 and 10 Hz. The major difference is that “high theta states” involve altering the dynamics of the circuit (Buzsáki et al., 2013), perhaps via increasing the amount of activity that is reverberating as well as improving the coordination of active volleys (Wiener, 1965; Strogatz, 1994).

In line with these ideas, the current data strongly support the theory that theta (a high power, low-frequency band) is a global rhythm while gamma is a local oscillation that gains strength from theta paced excitatory input. The theta-theta coupling between the CA1 region and anterior thalamus was larger than cross-regional theta-gamma coupling, supporting the idea that the 8 Hz activity is primarily related to excitation that “chases its own tail” within the Papez circuit (Vertes et al., 2001). Activity in the gamma band is a signature of excitatory-inhibitory interactions which tend to be more local (Buzsáki and Wang, 2012; Ray and Maunsell, 2015). As interneurons extensively populate the hippocampus (for review, see Klausberger and Somogyi, 2008), the smaller anatomic loops would lead to more cohesive dynamics accounting for a larger, faster gamma frequency relative to the anterior thalamus. To our knowledge, there are no reports of inhibitory interneurons that reside within the anterior thalamus (Wang et al., 1999). Rather, one plausible source of GABAergic input into the anterior thalamus arises from the reticular nucleus of the thalamus (Gonzalo-Ruiz and Lieberman, 1995; Lozsádi, 1995) and facilitates the neural activity that correlates to head direction (Vantomme et al., 2020). Projections out of the anterior thalamus “close the loop” via return projections to the entorhinal cortex, presubiculum, parasubiculum, and postsubiculum. Another possible mechanism for inhibitory input into the anterior thalamus is the hippocampus. An intriguing recent discovery has found a long-range inhibitory projection from CA3 to the anterior dorsal nucleus of the thalamus that modulates remote memory retrieval (Vetere et al., 2021). Thus, the reticular nucleus, CA3 inhibitory input, and the other potential sources of inhibitory input operate synergistically to maintain excitatory-inhibitory balance within the anterior thalamus. These inhibitory loops of the anterior thalamus are larger than the local inhibition-excitation loops of the hippocampus, capping the maximum frequency of anterior thalamic gamma for reasons such as a longer axonal conduction velocity (Fig. 3). Alternatively, the reticular thalamus input could be functioning to balance inhibition and excitation while the long-range hippocampal inhibitory input serves to globally coordinate interactions (Buzsáki et al., 2004). Of course, with biological systems, the truth could very well be somewhere in between (inhibition plays a role in both inhibitory-excitatory balance and cross-regional coordination simultaneously with a single physiological mechanism achieving both outcomes).

Broadly, these data support the “energy cascade” (Buzsáki and Draguhn, 2004) classical physics description of turbulence as applied to neuroscience (Sheremet et al., 2018a; Deco and Kringelbach, 2020). As described in Richardson’s simple poem “Big whorls have little whorls Which feed on their velocity,” in which the extension here is that the large anatomic reentrant loops that support theta comprise the “big whorls” which drive the anatomically smaller loops (little whorls). Previously, we have demonstrated that within the hippocampus, as theta amplitude increases, so does the amplitude and frequency of gamma (Sheremet et al., 2019). Here, we observed that gamma power correlates with theta power in the anterior thalamus as well. Moreover, although there was a nonsignificant trend of increased nonlinear phase coupling between theta and gamma within the anterior thalamus, there was an increase in theta-theta and theta-gamma nonlinear phase coupling between the CA1 region and the anterior thalamus. Therefore, it is plausible that the anterior thalamus is part of multiple, multiscale anatomic loops that propagate theta throughout the brain (Vertes et al., 2001). As gamma is local, it should be considered that any “cross-regional correlation” in frequency has more to do with the common theta drive than frequency-specific communication (Pernía-Andrade and Jonas, 2014; Ray and Maunsell, 2015). Gamma is the reverberatory consequence of common theta drive (Mizuseki et al., 2009; Zhou et al., 2022). Future research should work toward understanding how the dynamic process of forming patterns within and propagating across regions supports higher cognitive functions.

## Synthesis

Reviewing Editor: Michaël Zugaro, CNRS, Collège de France, Inserm Decisions are customarily a result of the Reviewing Editor and the peer reviewers coming together and discussing their recommendations until a consensus is reached. When revisions are invited, a fact-based synthesis statement explaining their decision and outlining what is needed to prepare a revision will be listed below. The following reviewer(s) agreed to reveal their identity: NONE.

The authors have drawn from a publicly available dataset and performed technically complex analyses, previously employed by the authors on similar data to useful and interesting effect. The analyses are sound and the results interesting, and most comments relate to the clarity of experimental motive and interpretation. The comments may at first appear numerous and lengthy, but this is only the result of an attempt to state as clearly as possible the issues raised during the review process. The manuscript has been judged favorably and mostly requires clarification.

Major issues

1) Perhaps the main issue is to clarify whether the scientific question, analyses and claims, apply to the entire anterior thalamus, or to one nucleus in particular.

The main challenge here is to test a hypothesis that is most obviously rationalized by general features of the anterior thalamus, with predictions most easily observed in rhythmically firing neurons, while working with a dataset that was collected mostly from a single anterior thalamic nucleus (AD), for the purpose of recording non-rhythmically firing neurons (HD cells). On one hand, it makes sense to discuss the anterior thalamic nuclei collectively because of their unique connective relationship as a group to limbic/parahippocampal structures. Still, on an individual basis, those connections are quite distinct among the three nuclei. Indeed, the parallel yet highly segregated mammillo-thalamic projections are a striking feature of the entire limbic system connective loop, as just one example. Experimental findings regarding neural activity or its interaction with other sites are also quite distinct among the three nuclei, including a major difference between AV and AM on the one hand and AD on the other, that seems quite relevant to the question at hand: the presence of strong theta-modulated firing in neurons both HD and otherwise in AV and AM, and its near complete absence in AD.

This confusion starts from the title that explicitly and specifically references AD, although some of the results may hold primarily for AV or AM. The Introduction is framed in the general context of the anterior thalamus, but often applies to AV/AM rather than to AD. For example, in the first paragraph appears the statement, “...the subiculum strongly projects to the anterior thalamus...potentially responsible for the entrainment of anterior thalamic neurons to theta...” but among the references for both of these assertions, anatomical and functional, none seem to support either entrainment to theta *in AD*, nor a clear candidate afferent projection that could promote it.

The distinctions among thalamic nuclei should be clarified, and the text and analyses should better indicate which claim/analysis applies to which nuclei. At least for spike train analyses, the anatomical location of individual cells can hopefully be recovered from the data, data description, or possibly directly from the authors.

2) Presumably, the analyses were restricted to data acquired during exploratory behavior. However, theta is also present during REM sleep, so it is possible that REM data were also analyzed. Could the authors clarify what the animals were doing in the epochs used for the analyses? If the behavior involved different task components, it may be worth processing shorter, functionally defined, episodes: for instance, gamma and theta oscillations vary with cognitive demands, across learning or task components (e.g. Igarashi et al. (2014), Montgomery et al. (2008)).

3) Head direction cells are further classified as high vs low firing cells. What is the rationale for this distinction? And why did the authors base the distinction on the time-averaged firing rate, rather than the most widely used peak firing rate? For readers familiar with the HD cell literature, groups labeled “fast” and “slow” which likely represent cells with low and high *peak* firing rates, respectively, is likely to be a recurrent source of confusion as they go through the paper. More importantly, a neuron, particularly one with a low background firing rate, whose primary firing correlate is head direction, will produce a time-averaged firing rate over the course of a recording session that is highly dependent on dwell time facing the preferred direction, which, in an open-field foraging task and given the typical motion statistics of freely-moving mice, may be well be brief.

At stake is disambiguation of a three-way confound: the theta/gamma X running speed effects could be a function of peak firing rate (a neuron/network property), time-averaged firing rate (a reflection of specific movement patterns), or sampling site (AD or elsewhere), with HD cells in this instance being more likely to also be AD cells, or of course some combination of these three. Since there seems to be some effect on HD cells that do not necessarily express theta modulation - which is interesting! - are there comparisons within the HD cell sample overall that could be made to identify the significant variable (e.g., peak firing rate/modulation depth, or power of theta modulation?) Incidentally, if the position of the recorded unit along the shank of the probe is available in the metadata, could it be combined with theta spectral power to identify AV neurons?

The main conclusion based on this distinction among HD cells is that the velocity modulation is stronger for high firing rate cells. Could this be tested more directly with a correlation or regression, rather than by splitting in two arbitrary groups?

Also, it is unclear how Figure 3 relates to the main topic of the paper (it does not examine velocity modulation). It is based on the possibly problematic time-averaged firing rate, and should somehow compensate for the critical confounding variable of head direction.

4) In Figure 4 (non-HD cells), there is a concern that the spectral peaks could be accounted for by the two most active cells. Can the authors rule out this possibility by confirming that the effect persists in the absence of these possible outliers? (As a side note, please remove the X-axis scale markings on the top 2 averaged PSD plots).

5) Please report the value of N for all tests. The authors acknowledge that the low statistical power may explain why some analyses are inconclusive. Presumably, in most analyses N is the number of mice: one solution may be to perform some statistics over sessions rather than animals, using a balanced number of sessions across animals (randomly subsampling if necessary).

6) A recent article claims that theta frequency is modulated by acceleration, not speed (Kropff et al. 2021). This contradicts the generally accepted role of speed as a modulatory signal, but the finding remains controversial. Although the dependent variable in the current manuscript is power, rather than frequency, it is possible that these are not entirely independent in terms of underlying mechanisms. Have the authors tested how acceleration impacts theta power? It may be interesting, at some point in the manuscript, to discuss how the current findings relate to the controversy about the nature of the signal (velocity vs acceleration) that modulates theta, possibly discussing the merits and shortcomings of the Kropff et al. study in light of any additional analyses that could be performed in the current study.

Minor Issues

Introduction

P4, 1st para, 1st sentence: “facilitating activity flow through the Papez circuit” may not be consistent with what Vantomme et al. (2020) report; indeed when the feedforward inhibition they describe is suppressed, the result is *increased* firing activity at the expense of spatial resolution.

P4, 1st para, generally: Central to the manuscript’s rationale is the energy cascade hypothesis proposed by Buzsaki and Draguhn, which relies on local circuits with interacting excitatory and inhibitory tone. Acknowledging the absence of inhibitory interneurons in AD, the authors point to GABA-ergic input from the thalamic reticular nucleus modulated by glutamatergic input from parahippocampal cortex, and to a newly-discovered sparse inhibitory projection from CA3 interneurons. Neither are particularly local relative to the larger theta-modulated circuit putatively driving them, nor are evidence of the kind of inhibitory drive that would likely support gamma oscillations (Vantomme et al, 2020 and Vetere et al, 2021 both demonstrate inhibitory modulation on AD under quite specific experimental conditions without any clear implications for LFP). It is hard to judge whether this presents a major problem to the energy cascade hypothesis, but the authors may refer to the following article that might provide a more satisfying solution to the local-inhibition problem: D Pinault, M Deschênes (1992) Voltage-dependent 40-Hz oscillations in rat reticular thalamic neurons in vivo. Neuroscience 51(2):245-58. doi:10.1016/0306-4522(92)90312-p.

Results

P7, 2nd para: ‘we observed an increase in frequency and power’ (and similar subsequent claims). Did the authors test for significance?

Very Minor Issues (copy-editing/style)

Abstract

1st sentence: “‘online’ state of the brain;” not clear what ‘online’ means here

Introduction

2nd para, 3rd sentence: turbulence is an illustrative analogy to what is proposed in the energy cascade hypothesis and I unreservedly support the use of quoted poetry in its service (and generally), but “if not being one and the same” is perhaps a step too far

Methods

Starting above equation (3) in ‘Time Series Analysis’ and in several other places further down, the superscripts in the bispectrum variable should be ppp (not ppq). In a couple instances, the word ‘quadrant’ should be replaced with ‘octant’.

Results

Figure 1. Labels in lower right panel seem to be reversed (AT<->CA1).

P9, last para, typo: “propr” should be “prior”

Figure 3 legend

Typo: modulation ‘deptd’ should be “depth”

Number agreement: “cells,” should be “cell,”

Figure 3 plot title: space needed between “Depth” and “vs”

P16, 2nd para, 2nd sentence: “Reorganization of the mouse hippocampal power spectra... with increases in running speed” Is an increase in spectral bands that already have higher power compared to others properly characterized as “reorganization?”

Same para, 5th sentence: sentence would parse more easily with a comma between “where” and “should”

P17, Limitations

“Our analyses may have been statistically underpowered”

It does not seem appropriate to raise in passing the thorny issues of p-hacking or the shortcomings of inferential statistics. Nor does it seem necessary: the failure of the trend in bicoherence coupling to reach significance in this instance is not a big threat to the general take-away here.

P17, next para: “On the contrary, the nervous system evolved against the background to form dynamic spatio-temporal patterns...” What background is being referred to here?

Figure labels & legends

There are quite a few instances of small typos, particularly missing spaces, among the figure labels and legends. A good hunt for these through the next version of the manuscript would be worthwhile.

## Author Response

Synthesis Statement for Author (Required):

The authors have drawn from a publicly available dataset and performed technically complex analyses, previously employed by the authors on similar data to useful and interesting effect. The analyses are sound and the results interesting, and most comments relate to the clarity of experimental motive and interpretation. The comments may at first appear numerous and lengthy, but this is only the result of an attempt to state as clearly as possible the issues raised during the review process. The manuscript has been judged favorably and mostly requires clarification.

We appreciate the diligence of the authors and editor as well as the review process afforded by eNeuro.

Major issues

1) Perhaps the main issue is to clarify whether the scientific question, analyses and claims, apply to the entire anterior thalamus, or to one nucleus in particular.

The main challenge here is to test a hypothesis that is most obviously rationalized by general features of the anterior thalamus, with predictions most easily observed in rhythmically firing neurons, while working with a dataset that was collected mostly from a single anterior thalamic nucleus (AD), for the purpose of recording non-rhythmically firing neurons (HD cells). On one hand, it makes sense to discuss the anterior thalamic nuclei collectively because of their unique connective relationship as a group to limbic/parahippocampal structures. Still, on an individual basis, those connections are quite distinct among the three nuclei. Indeed, the parallel yet highly segregated mammillo-thalamic projections are a striking feature of the entire limbic system connective loop, as just one example. Experimental findings regarding neural activity or its interaction with other sites are also quite distinct among the three nuclei, including a major difference between AV and AM on the one hand and AD on the other, that seems quite relevant to the question at hand: the presence of strong theta-modulated firing in neurons both HD and otherwise in AV and AM, and its near complete absence in AD.

This confusion starts from the title that explicitly and specifically references AD, although some of the results may hold primarily for AV or AM. The Introduction is framed in the general context of the anterior thalamus, but often applies to AV/AM rather than to AD. For example, in the first paragraph appears the statement, “...the subiculum strongly projects to the anterior thalamus...potentially responsible for the entrainment of anterior thalamic neurons to theta...” but among the references for both of these assertions, anatomical and functional, none seem to support either entrainment to theta *in AD*, nor a clear candidate afferent projection that could promote it.

The distinctions among thalamic nuclei should be clarified, and the text and analyses should better indicate which claim/analysis applies to which nuclei. At least for spike train analyses, the anatomical location of individual cells can hopefully be recovered from the data, data description, or possibly directly from the authors. We implemented a unique approach to recover location information from HD cell distribution versus theta-phase (TP) cell distribution across probes, see Figure 2 in the paper. (Note that we know the probe number that each cell belongs to, but we don’t know the depth of each cell from the spike sorting result.)

The channels we adopted for the LFP figures are from the probes with highest density of HD cells and highest density of theta phase modulated cells respectively. i.e.,

M12: ADT-Probe 4; AVT-Probe 7.

M17: ADT-Probe 6; AVT-Probe 8.

M20: ADT-Probe 3; AVT-Probe 5.

M32: ADT-Probe 4; AVT-Probe 7.

Furthermore, we also collected as much information as possible regarding the Electrodes’ location information, see citations below: For histology details from citations see below:

(Cited from Adrien et al. 2015) Thalamic probes were implanted in the left hemisphere, perpendicularly to the midline, (AP: -0.6 mm; ML: -0.5 to -1.9 mm; DV: 2.2 mm), with a 10 - 15{degree sign} angle, the shanks pointing toward midline (see Supplementary Fig. 1a-f).

Hippocampal wire bundles were implanted above CA1 (AP: -2.2 mm; -1 to -1.6 mm ML; 1 mm DV).

(Cited from CRCNS) The data set contains recordings made from multiple anterior thalamic nuclei, mainly the anterodorsal (AD) nucleus... in freely moving mice. Thalamic and subicular electrodes yielding high number of the so-called Head-Direction (HD) cells were likely to be located in the AD nucleus and the PoS, respectively. Electrode placement was confirmed by histology.

(Cited from CRCNS) ...In the four other animals [this refers to the four animals we were using in the current manuscript], electrodes were implanted in the anterior thalamus and in the pyramidal layer of the CA1 area of the hippocampus but only to record Local field Potentials (LFPs).

(Cited from CRCNS) Anterior Thalamus: 8 shank probe, Neuronexus Buz64 design.

Shank #1 is the most lateral, #8 the most medial Probe is perpendicular to the midline (coronal plane), tilted by 15 degrees (tips pointed toward midline), mounted on a movable drive. Insertion coordinates: AP -0.7mm, ML 0.5mm Approx depth from surface:

2.97mm.

(Cited from CRCNS) Hippocampus: 5 tungsten wires (fifty micrometer in diamater), one (at least) is in the pyramidal layer of CA1 as shown by the presence of Sharp Wave/Ripples. Insertion coordinates: AP -2.2mm, ML 1.3mm, depth: 1.7mm (Cited from Adrien et al. 2015) ...We addressed the contributions of stimulus-driven and internally-generated activity by recording ensembles of HD neurons in the antero-dorsal thalamic nucleus and the postsubiculum of mice by comparing their activity in various

brain states. ...

(Cited from Adrien et al. 2015) a: Dual site recording of cell ensembles in the Antero-Dorsal nucleus of the thalamus (ADn) and the post-subiculum (PoS). 4′ ,6-Diamidino-2-Phenylindole (DAPI) staining of a coronal section through the PoS (top; arrowheads indicate tracks) and ADn (bottom; DAPI combined with parvalbumin yellow fluorescent protein, PV-YFP). RT, reticular nucleus.

a-c: Histology. a: 4’,6-Diamidino-2-Phenylindole (Dapi) staining of a thalamic slice. Three electrode tracks are visible in this section. b: Fluorescent photograph of PV-YFP showing high-density parvalbumin immunoreactivity signal in the reticular nucleus of the thalamus. c: Merged picture with labeled nuclei. AD, anterodorsal; LD, laterodorsal; AV, anteroventral, AM, anteromedial, MD, mediodorsal; Re, reuniens, RT reticular nucleus. d-f: Localization of HD cells. d: fraction of HD cells recorded by two adjacent shanks. Session #1 (top squares) corresponds to the first session which detected units in the thalamus. The probe was lowered by 70-140 mm at the end of each session. e: Interpolated density of HD cells, based on known inter-shank spacing and the amount of incremental movement of the recording sites between each session. Brightness codes for total number of clustered HD units. f: Putative anatomical density of HD cells superimposed on mouse brain atlas (Allen Mouse Brain Atlas.

Available from: http://mouse.brain-map.org). g: Same as a for a slice including the post-subiculum. Arrowheads show four out of the six electrode tracks. PoS: post-subiculum; PRE: pre-subiculum; SUB: subiculum; RSP: retrosplenial cotex; V1: primary visual cortex; SC: superior colliculus. h: same as f for the animal shown in g.

2) Presumably, the analyses were restricted to data acquired during exploratory behavior. However, theta is also present during REM sleep, so it is possible that REM data were also analyzed. REM analysis has been added, see updated Figure 1, 6, 7, 8, 9, 10 and 11 in the manuscripts. Could the authors clarify what the animals were doing in the epochs used for the analyses? If the behavior involved different task components, it may be worth processing shorter, functionally defined, episodes: for instance, gamma and theta oscillations vary with cognitive demands, across learning or task components (e.g. Igarashi et al. (2014), Montgomery et al. (2008)).

As far as we can see, we have sessions for M17, M20, M32 in an open environment. For M12, at the data processing stage, we did take into account of the session of exploration in a radial maze (only foraging for food, with no specific cognitive tasks), and merged together the data with exploration in the open environment. Also, see citations of the behavior information below:

(Cited from Adrien et al. 2015) Recording sessions were composed of exploration of an open environment (‘wake’ phase) during which the animals were foraging for food for 30 to 45 minutes. The environment was a 53 × 46 cm rectangular arena surrounded by 21-cm high, black-painted walls on which were displayed two salient visual cues. The exploration phase was (in 41 out of 42 sessions) preceded and followed by sleep sessions of about 2 hours duration. In one session, the exploration of a radial maze following the second sleep phase was considered as the ‘wake’ period and no sleep was recorded subsequently. All experiments were carried out during daylight in normal light dark cycle. (Cited from Adrien et al. 2015) Ensembles of HD neurons from ADn (8.4 {plus minus} 5.1 s.d. units per session) and PoS (5 {plus minus} 2.8 s.d.) were recorded by multi-site silicon probes (Fig. 1a, Supplementary Fig. 1) in 7 mice foraging for food in an open environment (42 sessions) and in their home cages during sleep. (Cited from Adrien et al. 2015) ... neuronal ensembles were monitored during sleep sessions (5 hours {plus minus}1 s.d.), before and/or after active exploration in the open field. Sleep stages were classified as Slow-Wave Sleep (SWS) or Rapid Eye Movement Sleep (REM) based on the animal’s movement and time-resolved spectra of the local field potential recorded from the hippocampus or the PoS...

3) Head direction cells are further classified as high vs low firing cells. What is the rationale for this distinction? And why did the authors base the distinction on the time-averaged firing rate, rather than the most widely used peak firing rate? For readers familiar with the HD cell literature, groups labeled “fast” and “slow” which likely represent cells with low and high *peak* firing rates, respectively, is likely to be a recurrent source of confusion as they go through the paper. More importantly, a neuron, particularly one with a low background firing rate, whose primary firing correlate is head direction, will produce a time-averaged firing rate over the course of a recording session that is highly dependent on dwell time facing the preferred direction, which, in an open-field foraging task and given the typical motion statistics of freely-moving mice, may be well be brief.

We agree that we should strive to maintain convention with the prior literature. Therefore, we modified the classification into 3 categories: 1. HD cells; 2. TP cells; 3. Other cells. Following the new classification, we did a new Figure 5 in the paper. The figures below show a comparison between peak firing rate and averaged firing rate versus HD modulation depth for the 4 mice, qualitatively they are similar in trend. Nevertheless, we replaced averaged firing rate by peak firing rate for the Figure 5 in the manuscript.

At stake is disambiguation of a three-way confound: the theta/gamma X running speed effects could be a function of peak firing rate (a neuron/network property), time-averaged firing rate (a reflection of specific movement patterns), or sampling site (AD or elsewhere), with HD cells in this instance being more likely to also be AD cells, or of course some combination of these three. Since there seems to be some effect on HD cells that do not necessarily express theta modulation - which is interesting! - are there comparisons within the HD cell sample overall that could be made to identify the significant variable (e.g., peak firing rate/modulation depth, or power of theta modulation?) Incidentally, if the position of the recorded unit along the shank of the probe is available in the metadata, could it be combined with theta spectral power to identify AV neurons?

We believe that the reviewer is expressing concerns about HD cells versus AV neurons. We have now estimated AD and AV locations by plotting the HD cell concentration versus TP cell concentration, see Figure 2 in the paper. The subregion in anterior thalamus that features high density of head direction cells is estimated as AD, while the subregion that features high density of theta phase cells (cells show strong modulation by theta phase) is estimated as AV.

The main conclusion based on this distinction among HD cells is that the velocity modulation is stronger for high firing rate cells. Could this be tested more directly with a correlation or regression, rather than by splitting in two arbitrary groups?

We now classify the cells into HD modulated, theta-phase modulated and non-modulated cells. It seems that the velocity modulation is stronger for theta-phase modulated cells over the other 2 groups，which can be seen form the Figure 5 in the paper. In addition, we split the velocities into three groups instead of only two.

Also, it is unclear how Figure 3 relates to the main topic of the paper (it does not examine velocity modulation). It is based on the possibly problematic time-averaged firing rate, and should somehow compensate for the critical confounding variable of head direction.

The possibly problematic time-averaged firing rate was replaced by peak firing rate in Figure 5. After the modifications, Figures 5 and 6 together indicate that HD and theta-phase modulation are likely uncorrelated, and theta-phase cells are the major contributors to the increase of rhythmicity and overall power versus velocity. 4) In Figure 4 (non-HD cells), there is a concern that the spectral peaks could be accounted for by the two most active cells. Can the authors rule out this possibility by confirming that the effect persists in the absence of these possible outliers? (As a side note, please remove the X-axis scale markings on the top 2 averaged PSD plots). The old Figure 4 was replaced by the new Figure 6 in the current revised manuscript.

However, since the review is concerned with the outliers, even though the old figure will not go into the paper, we added the PSD of the 2 outliers into the figure together with the PSD without the outliers, to directly address the reviewer’s comment: 5) Please report the value of N for all tests. The authors acknowledge that the low statistical power may explain why some analyses are inconclusive. Presumably, in most analyses N is the number of mice: one solution may be to perform some statistics over sessions rather than animals, using a balanced number of sessions across animals (randomly subsampling if necessary).

We have made certain that the DOFs are reported in our statistics. For clarification, we run on stats on a per animal basis (which was a total of 4 mice). While we appreciate the Reviewer’s suggestion of using sessions as sample size rather than mice, respectfully, we do not feel that it is appropriate to run the analyses in that way. Specifically, using the number sessions violates the statistical assumption of independence of samples. Data from the same mouse are not independent samples. 6) A recent article claims that theta frequency is modulated by acceleration, not speed (Kropff et al. 2021). This contradicts the generally accepted role of speed as a modulatory signal, but the finding remains controversial. Although the dependent variable in the current manuscript is power, rather than frequency, it is possible that these are not entirely independent in terms of underlying mechanisms. Have the authors tested how acceleration impacts theta power? It may be interesting, at some point in the manuscript, to discuss how the current findings relate to the controversy about the nature of the signal (velocity vs acceleration) that modulates theta, possibly discussing the merits and shortcomings of the Kropff et al. study in light of any additional analyses that could be performed in the current study.

We recently published a rebuttal to the Kropff et al. (2021) report (Kennedy et al., 2022; PMID: 35477905). Specifically, we analyzed our own data from freely behaving rats as well as data from the Moser Lab speed-clamping experiment. Importantly, the analyses conducted by Kropff et al., (2022) failed to account for the inter-dependence of acceleration and speed. We therefore analyzed the contribution of both speed and acceleration to the variance in theta frequency and power in tandem. In unrestrained behavior, speed remains the dominant behavioral correlate to theta amplitude and frequency. Positive acceleration in the speed-clamp experiment induced a transient increase in theta frequency and power. However, speed retained the dominant influence over theta frequency, changing with velocity in both acceleration and deceleration conditions.

Minor Issues

Introduction

P4, 1st para, 1st sentence: “facilitating activity flow through the Papez circuit” may not be consistent with what Vantomme et al. (2020) report; indeed when the feedforward inhibition they describe is suppressed, the result is *increased* firing activity at the expense of spatial resolution.

We have now clarified this “ facilitating the timing of activity flow through the Papez circuit (Vantomme et al., 2020)”. P4, 1st para, generally: Central to the manuscript’s rationale is the energy cascade hypothesis proposed by Buzsaki and Draguhn, which relies on local circuits with interacting excitatory and inhibitory tone. Acknowledging the absence of inhibitory interneurons in AD, the authors point to GABA-ergic input from the thalamic reticular nucleus modulated by glutamatergic input from parahippocampal cortex, and to a newly-discovered sparse inhibitory projection from CA3 interneurons. Neither are particularly local relative to the larger theta-modulated circuit putatively driving them, nor are evidence of the kind of inhibitory drive that would likely support gamma oscillations (Vantomme et al, 2020 and Vetere et al, 2021 both demonstrate inhibitory modulation on AD under quite specific experimental conditions without any clear implications for LFP). It is hard to judge whether this presents a major problem to the energy cascade hypothesis, but the authors may refer to the following article that might provide a more satisfying solution to the local-inhibition problem: D Pinault, M Deschênes (1992) Voltage-dependent 40-Hz oscillations in rat reticular thalamic neurons in vivo. Neuroscience 51(2):245-58. doi:10.1016/0306- 4522(92)90312-p. We have included the sentence “ One potential source of fast local rhythmicity may be a voltage dependent oscillation as seen in the reticular thalamus (Pinault and Deschênes, 1992).” Results P7, 2nd para: ‘we observed an increase in frequency and power’ (and similar subsequent claims). Did the authors test for significance? Figure 3 now includes a comparison using confidence intervals. Very Minor Issues (copy-editing/style)

Abstract

1st sentence: “‘online’ state of the brain;” not clear what ‘online’ means here We have now removed “online”.

Introduction

2nd para, 3rd sentence: turbulence is an illustrative analogy to what is proposed in the energy cascade hypothesis and I unreservedly support the use of quoted poetry in its service (and generally), but “if not being one and the same” is perhaps a step too far We removed the offending words. We do, however, encourage the reviewer to explore “Energy Cascade” on Wikipedia and make their own inferences regarding the nuanced differences between it and turbulence (https://en.wikipedia.org/wiki/Energy_cascade). Perhaps they will find them quite similar.

Methods

Starting above equation (3) in ‘Time Series Analysis’ and in several other places further down, the superscripts in the bispectrum variable should be ppp (not ppq). In a couple instances, the word ‘quadrant’ should be replaced with ‘octant’. Sheremet et al. (2016; 2019) were using the notation ppq for cross-bispectrum, and the bispectrum is a particular case, an “auto” version of the cross-bispectrum, with p ≡ q. It is not necessary to change the notations to qqq for all math related to the “auto” Bispectrum for the reader to have in mind that the “auto” version is a subclass of the “cross” version and keeping the notation ppq seems to be more general.

Results

Figure 1. Labels in lower right panel seem to be reversed (AT<->CA1). The reviewer is correct. The Figure 1 was updated: P9, last para, typo: “propr” should be “prior” Fixed. Thank you. Figure 3 legend Typo: modulation ‘deptd’ should be “depth” Number agreement: “cells ,” should be “cell,” Figure 3 plot title: space needed between “Depth” and “vs” Thank you. These changes have been made. P16, 2nd para, 2nd sentence: “Reorganization of the mouse hippocampal power spectra... with increases in running speed” Is an increase in spectral bands that already have higher power compared to others properly characterized as “reorganization?” This now reads “ Complimenting research from the rat hippocampus, we found that there is a redistribution of power in the mouse hippocampal power spectra with a decrease in low frequencies (∼1-4 Hz) and an increase in theta, theta harmonic, and gamma power with increases in running speed.” Same para, 5th sentence: sentence would parse more easily with a comma between “where” and “should” Corrected. Thank you P17, Limitations“ Our analyses may have been statistically underpowered” It does not seem appropriate to raise in passing the thorny issues of p-hacking or the shortcomings of inferential statistics. Nor does it seem necessary: the failure of the trend in bicoherence coupling to reach significance in this instance is not a big threat to the general take-away here. We have now rephrased this to read “ However, a downside of this is that, while the approach and animal number were optimized by the initial researchers, our study may have benefited from implementing other behavioral tests (e.g., mnemonic tests), more animals, or different electrode configurations. “

P17, next para: “On the contrary, the nervous system evolved against the background to form dynamic spatio-temporal patterns...” What background is being referred to here? We have rewritten this sentence to read “ On the contrary, the nervous system evolved against the selective pressure of rapid environmental changes (i.e., “anticipate” what happens next and respond appropriately).” Figure labels & legends There are quite a few instances of small typos, particularly missing spaces, among the figure labels and legends. A good hunt for these through the next version of the manuscript would be worthwhile. We appreciate this and have gone through the manuscript using ‘Grammarly’. We hope to have captured these instances of missing typos.
